# Adherence to CONSORT Guidelines and Reporting of the Determinants of External Validity in Clinical Oncology Randomized Controlled Trials: A Review of Trials Published in Four Major Journals between 2013 and 2015

**DOI:** 10.3390/curroncol30020160

**Published:** 2023-02-08

**Authors:** Sophie Audet, Catherine Doyle, Christopher Lemieux, Marc-Antoine Tardif, Andréa Gauvreau, David Simonyan, Hermann Nabi, Julie Lemieux

**Affiliations:** 1 Département d’Hématologie, Hôpital de Chicoutimi, Chicoutimi, QC G7H 5H6, Canada; 2 Département d’Hématologie, Centre Hospitalier Universitaire (CHU) de Québec—Université Laval, Québec, QC G1R 2J6, Canada; 3 Faculté de Médecine, Université Laval, Québec, QC G1V 0A6, Canada; 4 Centre de Recherche du CHU de Québec—Université Laval, Québec, QC G1V 4G2, Canada; 5 Département d’Hémato-oncologue, Hôpital de Rimouski, Rimouski, QC G5L 5T1, Canada

**Keywords:** external validity, CONSORT, randomized trials, oncology

## Abstract

Our primary objective was to determine the proportion of trials that report the number of patients assessed for eligibility before randomization. We performed the systematic retrieval and analysis of all phase II, III, and IV RCTs published between 2013 and 2015 in four high-impact-factor journals in the field of clinical oncology. Among 456 RCTs reviewed, 236 trials (51.8%) reported the number of patients assessed for eligibility. Among the 236 trials that reported the entire enrollment process, the reasons for patient exclusion could be found in 184 trials (78%). A flow diagram was presented in 452 trials (99.1%), and 98 trials (21.5%) included a discussion on generalizability. Reporting the parameters of external validity in medical oncology RCTs is challenging. Improving adherence to the 2010 CONSORT guidelines concerning the enrollment process could help clinicians and health policymakers establish to whom trial results apply.

## 1. Introduction

The number of scientific research papers published yearly rapidly expands [1,2]. The Consolidated Standards of Reporting Trials (CONSORT) statement was developed by a group of biomedical journal editors to improve the quality of randomized controlled trial (RCT) reporting. It consists of a set of evidence-based recommendations published in 1996 and updated in 2010 [3,4]. The CONSORT statement has since been endorsed by the International Committee of Medical Journal Editors [5] and is recommended by the Enhancing the QUAlity and Transparency Of health Research (EQUATOR) network, which is an international initiative to improve the reliability and value of published health research literature [6]. Even though their recommendations are not enforced consistently [7], their adoption has translated into measurable improvements in many RCT quality criteria [8,9], including the method of sequence generation and allocation concealment. 

Nevertheless, the reporting of the enrolment process and the other determinants of external validity (also called generalizability) is challenging [10,11]. The assessment of external validity is of paramount importance as it allows clinicians to determine whether they can extrapolate findings from a study to the patients they care for in everyday practice. It can be effective only if details are provided about the participants of the study sample and the source population from which they were recruited [12].

Providing information on the enrollment process, particularly the difference between the number of patients screened for eligibility and those ultimately randomized, is crucial for several reasons. First, it estimates the proportion of all potentially eligible people who met the study requirements, which may indicate how stringent inclusion and exclusion criteria were applied to select participants. Second, it allows the detection of arbitrary exclusions, which may introduce selection biases and thus affect the representativeness of the participants included in the trial. Finally, it allows for knowing the number of participants who withdrew consent before randomization. Thus, details of the enrollment process are relevant to assess the generalizability of trial findings and optimize recruitment to RCTs by helping to identify potential obstacles to accrual.

**Figure 1:**
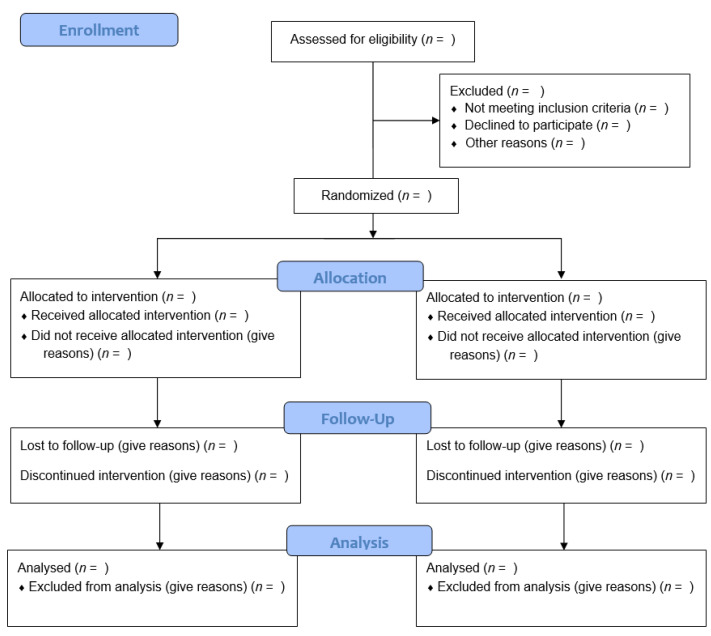
Template CONSORT flow diagram of the progress through the phases of a parallel randomized controlled trial of two groups (that is, enrolment, intervention allocation, follow-up, and data analysis).

The CONSORT statement, updated in 2010, makes several recommendations pertaining to external validity (Table 1). Specifically, the flow of participants throughout the study, from enrollment to analysis, should be detailed, and it is strongly recommended to present it in diagram form (Figure 1). The number of patients assessed for eligibility should be stated in the diagram, along with the reasons for patient exclusion. A discussion on the generalizability of the trial findings should also be included.

This review was performed to evaluate the adherence to CONSORT guidelines concerning the enrollment process and other external validity determinants in medical oncology RCTs. Our objectives were to determine the proportion of trials that report the number of patients assessed for eligibility before randomization and to identify variables affecting this reporting. We further aimed to determine the proportion of trials that state the reasons for patient exclusion and include a flow diagram and a discussion on the generalizability of the findings.

## 2. Materials and Methods

### 2.1. Study Design and Trial Selection

We performed a systematic retrieval and analysis of all phase II, III, and IV RCTs published 3 to 5 years after the publication of the updated version of the CONSORT statement in 2010 (i.e., January 2013 and December 2015) in the field of clinical oncology and four high-impact-factor peer-reviewed journals: *Journal of Clinical Oncology*, *Journal of the National Cancer Institute*, *Lancet Oncology*, and *The New England Journal of Medicine*.

One reviewer (SA) screened all titles and abstracts for relevance. The exclusion criteria were (1) studies presenting sub-group analyses, secondary endpoints, or follow-up studies; (2) studies presenting interim results; (3) studies reporting results from multiple studies simultaneously; (4) studies investigating non-therapeutic interventions; or (5) studies in which the unit of randomization was not individual patients.

### 2.2. Data Extraction

For each paper, data were extracted by one reviewer (SA) and compared to data independently extracted by a second reviewer (CD, CL, M-AT, AG, or JL) using a standardized form (Supplementary Material File S1). Discrepancies were resolved by consensus between SA and JL and by a third reviewer when no consensus was reached between SA and JL. The following information was recorded: journal, date of paper publication, cancer type, cancer stage, intervention type, study phase, sponsor type and name, mention of the number of patients assessed for eligibility or screened in the main article or the supplementary appendix, number of patients assessed for eligibility or screened, number of patients enrolled or registered, number of patients excluded before randomization and number of patients randomized, reasons for patient exclusion with the numbers of patients for each reason, inclusion of a flow diagram (whether or not the section on eligibility was there) in the main article or the supplementary appendix, whether the trial had a positive or negative outcome, and presence of a discussion on the generalizability of the trial findings.

### 2.3. Definition of Trial Characteristics

For this review, cancer stages were defined as follows: localized was equivalent to stage I, locally advanced was equivalent to stages II and III, and advanced was equivalent to stage IV or recurrent cancer. For hematologic cancers, the stage was other/hematologic cancer. A trial was considered positive when the investigational treatment was shown to be superior (or non-inferior in the case of non-inferiority studies) to the standard treatment. The presence of a discussion on the generalizability of the trial findings was assessed by reviewing the discussion section of the articles.

### 2.4. Statistical Analyses

Descriptive statistics included the frequency and proportion estimations for categorical factors and median and interquartile ranges for continuous variables. The inter-rater agreement between the reviewers was estimated using simple and weighted Cohen’s kappa coefficients for categorical factors and intra-class correlation coefficients for continuous variables. Bivariate analysis included Pearson’s chi-square test or Fisher’s exact test when appropriate. Cross-tabulation and tests on proportions were performed between the trials that reported the number of patients assessed for eligibility before randomization and the following variables: journal, year of publication, cancer type, cancer stage, intervention type, study phase, and sponsor type. Bivariate log-binomial models were fitted to compare the pairwise proportions between these groups using Tukey–Kramer adjusted *p*-values. Cross-tabulation and tests on proportions were also performed between trials, including a discussion on the generalizability of the findings and the following variables: study phase and whether the trial was positive or negative. Statistical analyses were performed using SAS Statistical Software v.9.4 (SAS Institute, Cary, NC, USA) with a two-sided significance level set at *p* < 0.05.

## 3. Results

**Figure 2:**
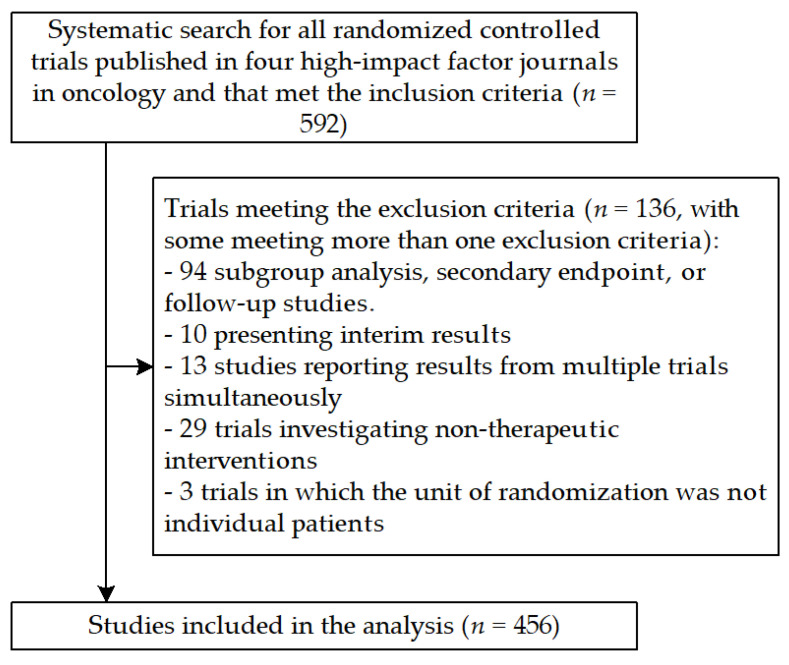
Flow diagram of the selection of randomized controlled trials in the review.

A total of 592 RCTs met the inclusion criteria. Of these, 136 trials were excluded due to meeting at least one exclusion criterion (Figure 2). The characteristics of the 456 included trials are presented in Table 2. The 456 included studies are listed in Supplementary Material File S2. Among the included trials, 228 (50%) were published in *Journal of Clinical Oncology*, 10 (2.2%) were published in *Journal of the National Cancer Institute*, 167 (36.6%) were published in *Lancet Oncology*, and 51 (11.2%) were published in *The New England Journal of Medicine*. About one-third of the trials were published each year from 2013 to 2015. The distribution of cancer/disease types is described in Table 2, with each of the main cancer categories representing 10%-15% of the trials. About half of the solid cancer stages were advanced. More than two-thirds of the studies were phase III and/or IV. Industry funding was present in 66.2% of the trials. The median sample size was 364 patients, ranging between 31 and 7576 participants.

The inter-rater agreement between reviewers (Table 3) was almost perfect (Cohen’s kappa = κ ≥ 0.81) for most variables, except for the discussion on the generalizability of trial findings (κ = 0.49). For the study phase “not found” (κ = 0.58) and the sponsor types “none” (κ = 0.5) and “not found” (κ = 0.45), the inter-rater agreement was moderate. This is due to the small number of trials in these categories and the fact that some reviewers did not look in other resources (e.g., www.clinicaltrials.gov, accessed date 25 January 2023) for missing information. 

The number of patients assessed for eligibility before randomization was reported in the main article in 219 trials, while 17 trials reported this information in the supplementary appendix, for a total of 236 trials, representing a proportion of 51.8% (236/456 trials). This proportion did not vary in a statistically significant manner with the cancer stage and did not increase over time, but it varied significantly between journals, cancer type, intervention type, study phase, and sponsor type, even though the absolute number of trials in each sub-group for cancer type and intervention type was small (Table 4).

Among the 236 trials that reported the proportion of patients assessed for eligibility, 78% (184/236 trials) mentioned the reasons for patient exclusion. The reasons for patient exclusion were “not meeting inclusion criteria” in 73.7% (174/236 trials), declining to participate or withdrawing consent in 59.3% (140/236 trials), death in 14.4% (34/236 trials), adverse events in 14.4% (34/236 trials), loss to follow-up in 5.9% (14/236 trials), and other reasons in 49.2% (116/236 trials). A total of 422 trials included a flow diagram in the main article, and an additional 30 trials included the diagram in the supplementary appendix, for a total of 452 trials, representing 99.1% (452/456) of trials. Finally, 21.5% (98/456) trials included a discussion on the generalizability of the results. This proportion was significantly higher among phase III and/or IV trials compared to phase II trials (85.2% (69/81 trials) vs. 14.8% (12/81 trials), p = 0.03). It was also higher among the trials that were considered positive compared to those considered negative (26.3% (67/255 trials) vs. 15.4% (31/201 trials), *p* = 0.005) (Table 5).

## 4. Discussion

Our review provides a systematic assessment of the reporting of external validity quality criteria, as defined by CONSORT guidelines, in RCTs in the field of medical oncology from influential journals. Even though 99% of the trials reported a flow diagram of patients, in half of them, the diagram started at the “randomization” stage. Thus, information on how many patients were screened for eligibility for the trial was missing. Among trials reporting eligible patients, almost one-quarter did not report the reasons for patient exclusion. A discussion on the generalizability of the findings was present in only 26% of the trials that reported positive results. 

Reviews that examined the determinants of external validity in various medical specialties have been found. In a report of 113 RCTs of healthcare interventions published in six major journals in 2004, 79% included a flow diagram, and 60% of those reported the number of patients assessed for eligibility, representing 47% of the total patient sample [10]. Another report of 469 RCTs indexed in PubMed core clinical journals in 2009 revealed that only 56% included a flow diagram, of which 81% reported the number of patients assessed for eligibility, or 45% of the total patient sample, with the reasons for patient exclusion mentioned in 60% of the trials [11]. Another review restricted to the field of medical oncology included 357 RCTs between 2005 and 2009 and showed that only 60% of these studies included a flow diagram [7]. Contrary to our findings, the results of this latter review suggest that the publication of the revised version of the CONSORT statement positively influenced the inclusion of the flow diagram of patient selection.

When retrospectively evaluating patients eligible for a clinical trial in cancer centers, it was found that 83.5% of patients fitting the main criteria in breast trials met all the eligibility criteria [13], and this number was 68% in hematological trials [14]. The recruitment fraction (number of patients recruited over the number potentially eligible) was 19.7% in breast trials and 23.1% in hematological trials. A study looking specifically at which eligibility criteria were a barrier to the recruitment of patients in the trial could not identify a unique category of eligibility criteria precluding enrollment as no shared specific eligibility criteria were reported by all trials as impeding enrolment [15]. 

It should be noted that the recommendation from CONSORT to provide details regarding the enrollment process is found in the suggested flow diagram but not in the checklist. Adding mandatory items related to the flow diagram in the checklist would probably help readers to evaluate whether the study population constitutes a highly selected subgroup and assess the risk of selection bias. In fact, as Palys and Berger explained [16], the CONSORT checklist is a subset of the Chalmers scale [17], which is more complete and stipulates the importance of detailing the entire enrolment process. 

The four journals we selected officially endorse the CONSORT statement and provide links to the CONSORT website in their “Instructions to authors”. One solution to improve adherence to the CONSORT statement would be to ask authors to complete the CONSORT checklist when submitting their manuscripts. Another solution would be to change the wording in the “Instructions to authors” to make adherence to the CONSORT statement mandatory rather than recommended, as Shamseer et al. suggested [18].

However, we recognize that it is challenging to collect these data, especially in large multicenter trials. Still, the reasons for screening failure are usually captured in large multicenter clinical trials, and the data should be made available. At least, the steps of the recruitment process should be detailed in the study protocol, and attempts should be made to implement them in a uniform manner across different centers and research professionals. The investigators should try to record the number of people identified as potentially eligible in the pre-screening, so that we can estimate the number of patients that need to be screened for every patient enrolled in the study. Nevertheless, the number of patients who have been offered participation in a trial by a physician and have declined to participate before being screened for eligibility would be difficult to collect in a meaningful way. The process of pre-screening is subject to selection bias. 

Our study has limitations. First, we evaluated data over 3 years (2013–2015) since we assume that a 3-year period would be reasonable to assess the changes after the update publication of the CONSORT guidelines in 2010, but we cannot exclude that reporting has evolved since 2016. Another limitation of our study is that the presence of a discussion on the generalizability of the trial findings is a subjective concept that depends on the reader’s judgment and the clinical context, as reflected by the poor inter-rater agreement coefficient between our reviewers. We were aware of this caveat, so we proceeded to a second reexamination of this variable when there was no consensus between the first two reviewers. To help readers interpret trial findings, a section entitled “To whom do the results of this trial apply?” in manuscripts, as suggested by Rothwell in 2005 [19], would be helpful. Lastly, there seemed to be confusion in the articles we reviewed between the terms “assessed for eligibility/screened” and “enrolled/registered”. The enrollment process of an RCT consists of three main steps; the first is to define a target population (i.e., a pre-screening in which the eligibility criteria guide the investigators to select the target population to approach for consent), the second is to screen the potential participants to determine their eligibility (the numbers of screened patients will always be equal to or larger than the numbers of enrolled patients), and the third is to invite eligible patients to enroll. The terms are not interchangeable; therefore, if a trial reported the number of patients “enrolled/registered”, we recorded it as such, even if it is possible that the authors meant “assessed for eligibility/screened”. We feel that this could be the object of clarification in the next revision of the CONSORT statement. The importance of the terms and steps of the enrollment process should be thoroughly emphasized, along with the importance of using them appropriately. Examining the numbers of excluded patients against the unmet inclusion criteria and the met exclusion criteria is crucial to the transparency and evaluation of the reliability of the enrollment process. Reporting the entire enrollment process should be mandatory. In addition, the recommendation from CONSORT to provide details regarding the enrollment process is found in the suggested flow diagram but not in the checklist. They should be added to the checklist.

The generalizability of the present study is limited to oncology trials. Nevertheless, they were published in four major journals in oncology that maintain high publication standards. All four selected journals are high-impact-factor journals publishing pivotal trials in oncology. Whether the present study findings could be generalizable to more modest journals and fields other than oncology remains unknown. Additional studies are necessary to examine these issues.

## 5. Conclusions

Our findings demonstrate that the reporting of some parameters of external validity of RCTs in medical oncology journals has been improved (e.g., presentation of the flow diagram), while others could be optimized in the future (e.g., reporting the numbers of patients assessed for eligibility). In order to facilitate the evaluation of the generalizability of trial results, investigators should be encouraged to collect and report data on all patients who consented to proceed with screening and subsequently enrolled if found eligible for the trial; if not enrolled, the reasons why they were not recruited should be reported as well. 

## Figures and Tables

**Table 1:** Quality of reporting of parameters of external validity from the 2010 CONSORT statement.

CONSORT Recommendation	Item Number in the CONSORT Checklist	Trials in Which the Item was Adequately Reported (*n* = 456)	
		*n*	%
Number of patients assessed for eligibility is indicated in the flow diagram	Not in the checklist, but included in the suggested flow diagram	236	51.8
Reasons for participant exclusion before randomization are detailed in the flow diagram, when applicable	Not in the checklist, but included in the suggested flow diagram	184	78 *
Flow diagram of participant flow throughout the study is included	13	452	99.1
Discussion on the generalizability of the trial findings is included	21	98	21.5

* The denominator for this proportion is 236, which is the number of trials that indicated the number of participants assessed for eligibility.

**Table 2:** Trial characteristics.

Characteristics	Studies (*n* = 456)	
	*n*	%
Journal		
Journal of Clinical Oncology	228	50
Journal of the National Cancer Institute	10	2.2
Lancet Oncology	167	36.6
The New England Journal of Medicine	51	11.2
Year of publication		
2013	159	34.9
2014	140	30.7
2015	157	34.4
Cancer type or category		
Breast	72	15.8
Central nervous system	11	2.4
Gastro-intestinal	65	14.2
Genito-urinary	50	11
Gynecological	20	4.4
Head and neck	14	3.1
Hematological	65	14.2
Melanoma	22	4.8
Lung	53	11.6
Sarcoma	8	1.8
Symptom control	57	12.5
Other	19	4.2
Cancer stage		
Localized	80	17.5
Locally advanced	57	12.5
Advanced	232	50.9
Other/Hematologic cancer	118	25.9
Intervention type		
Chemotherapy	89	19.5
Hormonotherapy	20	4.4
Immunotherapy	13	2.9
Radiotherapy	16	3.5
Surgery	10	2.2
Targeted therapy	181	39.7
Other	127	27.9
Study phase		
II	103	22.6
III/IV	317	69.5
Not found	40	8.8
Sponsor type (not mutually exclusive)		
Governmental/Academic/Foundation/Cooperative group	234	51.3
Pharmaceutical	303	66.4
None/not found	8	1.7
Trial outcome		
Positive	255	55.9
Negative	201	44.1
Sample size		
Median	364	
Range	31–7576	

**Table 3:** Correlation rates between reviewers.

Item	Cohen’s Kappa Coefficient *^,§^
Cancer type	0.99
Cancer stage	1
Intervention type	0.87
Study phase	
II	0.92
III/IV	0.84
Not found ^¥^	0.58
Sponsor type	
Governmental/Academic/Foundation/Cooperative group	0.90
Industrial	0.87
None ^¥^	0.50
Not found ^¥^	0.45
Number of patients assessed for eligibility specified in the flow diagram of the main article	0.90
If not in the main article, number of patients assessed for eligibility specified in the flow diagram of the supplementary appendix	1
Flow diagram included in the main article	0.97
If not in the main article, flow diagram included in the supplementary appendix	1
Discussion on the generalisability of the trial findings in the main text	0.49

* Simple kappa coefficients were used for binary variables, and weighted kappa coefficients were used for variables with more than two categories. ^§^ Interpretation of Cohen’s kappa (Landis JR & Koch GG, 1977): values of <0 indicate no agreement between observers, 0–0.20 indicates slight agreement, 0.21–0.40 indicates fair agreement, 0.41–0.60 indicates moderate agreement, 0.61–0.80 indicates substantial agreement, and 0.81–1 indicates almost perfect agreement. ^¥^ For the study phase and sponsor type items, the sub-optimal correlation rates might be attributable to the fact that some reviewers initially did not look for external sources of information outside of the articles under review, such as clinicaltrials.gov and other similar resources.

**Table 4:** Bivariate analyses of variables affecting the reporting of the number of patients assessed for eligibility.

Variable	Level	Trials Reporting the Number of Patients Assessed for Eligibility				*p*-Value *
		No = 220		Yes = 236		
		*n/N*	%	*n/N*	%	
Year	2013	84/159	52.8	75/159	47.2	0.3249
	2014	66/140	47.1	74/140	52.9	
	2015	70/157	44.6	87/157	55.4	
Journal	JCO **^a^**	124/228	54.4	104/228	45.6	0.0160 (Fisher)
	JNCI	7/10	70.0	3/10	30.0	
	LO **^a^**	67/167	40.1	100/167	59.9	
	NEJM	22/51	43.1	29/51	56.9	
Cancer type	Breast **^a^**	43/72	59.7	29/72	40.3	<0.0001 (Fisher)
	Central nervous system **^b^**	3/11	27.3	8/11	72.7	
	Gastro-intestinal **^c^**	26/65	40.0	39/65	60.0	
	Genito-urinary **^d^**	42/70	60.0	28/70	40.0	
	Head and neck	7/14	50.0	7/14	50.0	
	Leukemia and lymphoma **^b,c,e,f^**	39/53	73.6	14/53	26.4	
	Lung	23/53	43.4	30/53	56.6	
	Multiple myeloma	4/12	33.3	8/12	66.7	
	Melanoma	10/22	45.5	12/22	54.5	
	Other **^e^**	5/19	26.3	14/19	73.7	
	Symptom control **^a,d,f^**	14/57	24.6	43/57	75.4	
	Sarcoma	4/8	50.0	4/8	50.0	
Cancer stage	Localized	48/76	63.2	28/76	36.8	0.0518
	Locally advanced	13/26	50.0	13/26	50.0	
	Localized + locally advanced	¼	25.0	¾	75.0	
	Locally advanced + advanced	11/27	40.7	16/27	59.3	
	advanced	88/205	42.9	117/205	57.1	
	Other/hematologic	59/118	50.0	59/118	50.0	
Intervention type	Chemotherapy **^a,b,c^**	62/89	69.7	27/89	30.3	<0.0001 (Fisher)
	Hormone therapy	10/20	50.0	10/20	50.0	
	Immunotherapy **^a^**	4/13	30.8	9/13	69.2	
	Other **^b^**	49/127	38.6	78/127	61.4	
	Radiotherapy	12/16	75.0	4/16	25.0	
	Surgery	4/10	40.0	6/10	60.0	
	Targeted therapy **^c^**	79/181	43.6	102/181	56.4	
Study phase	II **^a^**	46/99	46.5	53/99	53.5	0.0023 (Fisher)
	III and/or IV^**b**^	163/313	52.1	150/313	47.9	
	II and III and/or IV	2/4	50.0	2/4	50.0	
	Not found **^a,b^**	9/40	22.5	31/40	77.5	
Sponsor type	Non industry	134/234	57.3	100/234	42.7	0.0001
	Pharmaceutical **^a^**	142/303	46.9	161/303	53.1	
	Not found	1/6	16.7	5/6	83.3	
	None	0/2	0	2/2	100	
	Non industry + Pharmaceutical **^a^**	57/220	25,9	32/236	13.6	

* Tests were based on Pearson’s Chi-squared test or Fisher’s exact test when applicable. **^a,b,c,d,e,f^** When the analysis (last column) was statistically significant (*p* < 0.05), all pairwise comparisons among groups were tested using the Tukey–Kramer adjusted *p*-value. Pairwise comparisons that were significantly different from one another are indicated by superscripts as follows: when the values for 2 groups do share a common superscript, they are significantly different (*p* < 0.05), whereas if the values do not share a common superscript, they are not significantly different.

**Table 5:** Bivariate analyses of variables affecting the reporting of a discussion on the generalizability of the trial findings.

Variable	Level	Trials Reporting a Discussion on Generalizability				*p*-Value *
		No		Yes		
		*n/N*	%	*n/N*	%	
Study phase ¤	II **^a^**	87/99	87.9	12/99	12.1	0.0039 (Fisher)
	III and/or IV **^b^**	244/313	78.0	69/313	22.0	
	II and III and/or IV	3/4	75.0	1/4	25.0	
	Not found **^a,b^**	24/40	60.0	16/40	40.0	
Trial outcome	Negative	170/201	84.6	31/201	15.4	0.0051
	Positive	188/255	73.7	67/255	26.3	

* Tests were based on Pearson’s Chi-squared test. ¤ After the exclusion of trials that were phase II and III at the same time and trials where the study phase was not found. **^a^**^,**b**^ When the analysis (last column) was statistically significant (*p* < 0.05), all pairwise comparisons among groups were tested using the Tukey–Kramer adjusted *p*-value. Pairwise comparisons that were significantly different from one another are indicated by superscripts as follows: when the values for 2 groups do share a common superscript, they are significantly different (*p* < 0.05), whereas if the values do not share a common superscript, they are not significantly different.

## Data Availability

Data are available from the corresponding author upon reasonable request.

## Supplementary Materials

The following supporting information can be downloaded at: https://www.mdpi.com/article/10.3390/curroncol30020160/s1, File S1: Standardized form used for data extraction. File S2: List of the revised reports in the present study.
