# Supplementary material for: Adherence to CONSORT Guidelines and Reporting of the Determinants of External Validity in Clinical Oncology Randomized Controlled Trials: A Review of Trials Published in Four Major Journals between 2013 and 2015
Source: Curr Oncol. 2023 Feb 8;30(2):2061–72. doi: 10.3390/curroncol30020160 (PMC9955486; doi:10.3390/curroncol30020160)
Supplement: Supplementary file 1 [file curroncol-30-00160-s001.zip › Curroncol-2121082 supplementary file S2 XML JCH.docx]

**Supplementary File 2.** List of the revised reports in the present study.

1. Abe T, Takeda K, Ohe Y, Kudoh S, Ichinose Y, Okamoto H, et al. Randomized phase III trial comparing weekly docetaxel plus cisplatin versus docetaxel monotherapy every 3 weeks in elderly patients with advanced non-small-cell lung cancer: the inter-group trial JCOG0803/WJOG4307L. J Clin Oncol. 2015;33:575-81.

2. Aebi S, Gelber S, Anderson SJ, Lang I, Robidoux A, Martin M, et al. Chemo-therapy for isolated locoregional recurrence of breast cancer (CALOR): a randomised trial. Lancet Oncol. 2014;15:156-63.

3. Ahn JS, Ahn YC, Kim JH, Lee CG, Cho EK, Lee KC, et al. Multinational Ran-domized Phase III Trial With or Without Consolidation Chemotherapy Using Docetaxel and Cisplatin After Concurrent Chemoradiation in Inoperable Stage III Non-Small-Cell Lung Cancer: KCSG-LU05-04. J Clin Oncol. 2015;33:2660-6.

4. Allegra CJ, Yothers G, O'Connell MJ, Beart RW, Wozniak TF, Pitot HC, et al. Neoadjuvant 5-FU or Capecitabine Plus Radiation With or Without Oxaliplatin in Rectal Cancer Patients: A Phase III Randomized Clinical Trial. J Natl Cancer Inst. 2015;107.

5. Allen JW, Moon J, Redman M, Gadgeel SM, Kelly K, Mack PC, et al. Southwest Oncology Group S0802: a randomized, phase II trial of weekly topotecan with and with-out ziv-aflibercept in patients with platinum-treated small-cell lung cancer. J Clin Oncol. 2014;32:2463-70.

6. Amadori D, Aglietta M, Alessi B, Gianni L, Ibrahim T, Farina G, et al. Efficacy and safety of 12-weekly versus 4-weekly zoledronic acid for prolonged treatment of pa-tients with bone metastases from breast cancer (ZOOM): a phase 3, open-label, random-ised, non-inferiority trial. Lancet Oncol. 2013;14:663-70.

7. Amadori S, Suciu S, Stasi R, Salih HR, Selleslag D, Muus P, et al. Sequential combination of gemtuzumab ozogamicin and standard chemotherapy in older patients with newly diagnosed acute myeloid leukemia: results of a randomized phase III trial by the EORTC and GIMEMA consortium (AML-17). J Clin Oncol. 2013;31:4424-30.

8. Andre F, O'Regan R, Ozguroglu M, Toi M, Xu B, Jerusalem G, et al. Everolimus for women with trastuzumab-resistant, HER2-positive, advanced breast cancer (BOLE-RO-3): a randomised, double-blind, placebo-controlled phase 3 trial. Lancet Oncol. 2014;15:580-91.

9. Andtbacka RH, Kaufman HL, Collichio F, Amatruda T, Senzer N, Chesney J, et al. Talimogene Laherparepvec Improves Durable Response Rate in Patients With Ad-vanced Melanoma. J Clin Oncol. 2015;33:2780-8.

10. Ang KK, Zhang Q, Rosenthal DI, Nguyen-Tan PF, Sherman EJ, Weber RS, et al. Randomized phase III trial of concurrent accelerated radiation plus cisplatin with or without cetuximab for stage III to IV head and neck carcinoma: RTOG 0522. J Clin Oncol. 2014;32:2940-50.

11. Angiolillo AL, Schore RJ, Devidas M, Borowitz MJ, Carroll AJ, Gastier-Foster JM, et al. Pharmacokinetic and pharmacodynamic properties of calaspargase pegol Esch-erichia coli L-asparaginase in the treatment of patients with acute lymphoblastic leuke-mia: results from Children's Oncology Group Study AALL07P4. J Clin Oncol. 2014;32:3874-82.

12. Araujo JC, Trudel GC, Saad F, Armstrong AJ, Yu EY, Bellmunt J, et al. Docetaxel and dasatinib or placebo in men with metastatic castration-resistant prostate cancer (READY): a randomised, double-blind phase 3 trial. Lancet Oncol. 2013;14:1307-16.

13. Ardeshna KM, Qian W, Smith P, Braganca N, Lowry L, Patrick P, et al. Rituxi-mab versus a watch-and-wait approach in patients with advanced-stage, asymptomatic, non-bulky follicular lymphoma: an open-label randomised phase 3 trial. Lancet Oncol. 2014;15:424-35.

14. Argiris A, Ghebremichael M, Gilbert J, Lee JW, Sachidanandam K, Kolesar JM, et al. Phase III randomized, placebo-controlled trial of docetaxel with or without gefitinib in recurrent or metastatic head and neck cancer: an eastern cooperative oncology group trial. J Clin Oncol. 2013;31:1405-14.

15. Arits AH, Mosterd K, Essers BA, Spoorenberg E, Sommer A, De Rooij MJ, et al. Photodynamic therapy versus topical imiquimod versus topical fluorouracil for treatment of superficial basal-cell carcinoma: a single blind, non-inferiority, randomised controlled trial. Lancet Oncol. 2013;14:647-54.

16. Awada A, Garcia AA, Chan S, Jerusalem GH, Coleman RE, Huizing MT, et al. Two schedules of etirinotecan pegol (NKTR-102) in patients with previously treated meta-static breast cancer: a randomised phase 2 study. Lancet Oncol. 2013;14:1216-25.

17. Bakitas MA, Tosteson TD, Li Z, Lyons KD, Hull JG, Li Z, et al. Early Versus De-layed Initiation of Concurrent Palliative Oncology Care: Patient Outcomes in the ENABLE III Randomized Controlled Trial. J Clin Oncol. 2015;33:1438-45.

18. Bang YJ, Im SA, Lee KW, Cho JY, Song EK, Lee KH, et al. Randomized, Dou-ble-Blind Phase II Trial With Prospective Classification by ATM Protein Level to Evaluate the Efficacy and Tolerability of Olaparib Plus Paclitaxel in Patients With Recurrent or Metastatic Gastric Cancer. J Clin Oncol. 2015;33:3858-65.

19. Barlesi F, Scherpereel A, Rittmeyer A, Pazzola A, Ferrer Tur N, Kim JH, et al. Randomized phase III trial of maintenance bevacizumab with or without pemetrexed after first-line induction with bevacizumab, cisplatin, and pemetrexed in advanced nonsqua-mous non-small-cell lung cancer: AVAPERL (MO22089). J Clin Oncol. 2013;31:3004-11.

20. Barrett-Lee P, Casbard A, Abraham J, Hood K, Coleman R, Simmonds P, et al. Oral ibandronic acid versus intravenous zoledronic acid in treatment of bone metastases from breast cancer: a randomised, open label, non-inferiority phase 3 trial. Lancet Oncol. 2014;15:114-22.

21. Barton DL, Liu H, Dakhil SR, Linquist B, Sloan JA, Nichols CR, et al. Wisconsin Ginseng (Panax quinquefolius) to improve cancer-related fatigue: a randomized, dou-ble-blind trial, N07C2. J Natl Cancer Inst. 2013;105:1230-8.

22. Baselga J, Gomez P, Greil R, Braga S, Climent MA, Wardley AM, et al. Ran-domized phase II study of the anti-epidermal growth factor receptor monoclonal antibody cetuximab with cisplatin versus cisplatin alone in patients with metastatic triple-negative breast cancer. J Clin Oncol. 2013;31:2586-92.

23. Batchelor TT, Mulholland P, Neyns B, Nabors LB, Campone M, Wick A, et al. Phase III randomized trial comparing the efficacy of cediranib as monotherapy, and in combination with lomustine, versus lomustine alone in patients with recurrent glioblas-toma. J Clin Oncol. 2013;31:3212-8.

24. Bath-Hextall F, Ozolins M, Armstrong SJ, Colver GB, Perkins W, Miller PS, et al. Surgical excision versus imiquimod 5% cream for nodular and superficial basal-cell car-cinoma (SINS): a multicentre, non-inferiority, randomised controlled trial. Lancet Oncol. 2014;15:96-105.

25. Beer TM, Armstrong AJ, Rathkopf DE, Loriot Y, Sternberg CN, Higano CS, et al. Enzalutamide in metastatic prostate cancer before chemotherapy. N Engl J Med. 2014;371:424-33.

26. Benboubker L, Dimopoulos MA, Dispenzieri A, Catalano J, Belch AR, Cavo M, et al. Lenalidomide and dexamethasone in transplant-ineligible patients with myeloma. N Engl J Med. 2014;371:906-17.

27. Bennouna J, Sastre J, Arnold D, Osterlund P, Greil R, Van Cutsem E, et al. Con-tinuation of bevacizumab after first progression in metastatic colorectal cancer (ML18147): a randomised phase 3 trial. Lancet Oncol. 2013;14:29-37.

28. Bepler G, Williams C, Schell MJ, Chen W, Zheng Z, Simon G, et al. Randomized international phase III trial of ERCC1 and RRM1 expression-based chemotherapy versus gemcitabine/carboplatin in advanced non-small-cell lung cancer. J Clin Oncol. 2013;31:2404-12.

29. Berry DL, Hong F, Halpenny B, Partridge AH, Fann JR, Wolpin S, et al. Elec-tronic self-report assessment for cancer and self-care support: results of a multicenter randomized trial. J Clin Oncol. 2014;32:199-205.

30. Bielack SS, Smeland S, Whelan JS, Marina N, Jovic G, Hook JM, et al. Metho-trexate, Doxorubicin, and Cisplatin (MAP) Plus Maintenance Pegylated Interferon Alfa-2b Versus MAP Alone in Patients With Resectable High-Grade Osteosarcoma and Good Histologic Response to Preoperative MAP: First Results of the EURAMOS-1 Good Re-sponse Randomized Controlled Trial. J Clin Oncol. 2015;33:2279-87.

31. Blay JY, Papai Z, Tolcher AW, Italiano A, Cupissol D, Lopez-Pousa A, et al. Ombrabulin plus cisplatin versus placebo plus cisplatin in patients with advanced soft-tissue sarcomas after failure of anthracycline and ifosfamide chemotherapy: a ran-domised, double-blind, placebo-controlled, phase 3 trial. Lancet Oncol. 2015;16:531-40.

32. Blay JY, Shen L, Kang YK, Rutkowski P, Qin S, Nosov D, et al. Nilotinib versus imatinib as first-line therapy for patients with unresectable or metastatic gastrointestinal stromal tumours (ENESTg1): a randomised phase 3 trial. Lancet Oncol. 2015;16:550-60.

33. Bonjer HJ, Deijen CL, Abis GA, Cuesta MA, van der Pas MH, de Lange-de Klerk ES, et al. A randomized trial of laparoscopic versus open surgery for rectal cancer. N Engl J Med. 2015;372:1324-32.

34. Borad MJ, Reddy SG, Bahary N, Uronis HE, Sigal D, Cohn AL, et al. Random-ized Phase II Trial of Gemcitabine Plus TH-302 Versus Gemcitabine in Patients With Ad-vanced Pancreatic Cancer. J Clin Oncol. 2015;33:1475-81.

35. Borghaei H, Paz-Ares L, Horn L, Spigel DR, Steins M, Ready NE, et al. Nivolumab versus Docetaxel in Advanced Nonsquamous Non-Small-Cell Lung Cancer. N Engl J Med. 2015;373:1627-39.

36. Bradley JD, Paulus R, Komaki R, Masters G, Blumenschein G, Schild S, et al. Standard-dose versus high-dose conformal radiotherapy with concurrent and consolida-tion carboplatin plus paclitaxel with or without cetuximab for patients with stage IIIA or IIIB non-small-cell lung cancer (RTOG 0617): a randomised, two-by-two factorial phase 3 study. Lancet Oncol. 2015;16:187-99.

37. Brahmer J, Reckamp KL, Baas P, Crino L, Eberhardt WE, Poddubskaya E, et al. Nivolumab versus Docetaxel in Advanced Squamous-Cell Non-Small-Cell Lung Cancer. N Engl J Med. 2015;373:123-35.

38. Breitbart W, Rosenfeld B, Pessin H, Applebaum A, Kulikowski J, Lichtenthal WG. Meaning-centered group psychotherapy: an effective intervention for improving psychological well-being in patients with advanced cancer. J Clin Oncol. 2015;33:749-54.

39. Bruera E, Hui D, Dalal S, Torres-Vigil I, Trumble J, Roosth J, et al. Parenteral hydration in patients with advanced cancer: a multicenter, double-blind, place-bo-controlled randomized trial. J Clin Oncol. 2013;31:111-8.

40. Bruera E, Yennurajalingam S, Palmer JL, Perez-Cruz PE, Frisbee-Hume S, Allo JA, et al. Methylphenidate and/or a nursing telephone intervention for fatigue in patients with advanced cancer: a randomized, placebo-controlled, phase II trial. J Clin Oncol. 2013;31:2421-7.

41. Bucaneve G, Micozzi A, Picardi M, Ballanti S, Cascavilla N, Salutari P, et al. Results of a multicenter, controlled, randomized clinical trial evaluating the combination of piperacillin/tazobactam and tigecycline in high-risk hematologic patients with cancer with febrile neutropenia. J Clin Oncol. 2014;32:1463-71.

42. Budd GT, Barlow WE, Moore HC, Hobday TJ, Stewart JA, Isaacs C, et al. SWOG S0221: a phase III trial comparing chemotherapy schedules in high-risk early-stage breast cancer. J Clin Oncol. 2015;33:58-64.

43. Buikhuisen WA, Burgers JA, Vincent AD, Korse CM, van Klaveren RJ, Schramel FM, et al. Thalidomide versus active supportive care for maintenance in patients with ma-lignant mesothelioma after first-line chemotherapy (NVALT 5): an open-label, multicentre, randomised phase 3 study. Lancet Oncol. 2013;14:543-51.

44. Burger JA, Tedeschi A, Barr PM, Robak T, Owen C, Ghia P, et al. Ibrutinib as In-itial Therapy for Patients with Chronic Lymphocytic Leukemia. N Engl J Med. 2015;373:2425-37.

45. Burnett AK, Russell NH, Hills RK, Hunter AE, Kjeldsen L, Yin J, et al. Optimi-zation of chemotherapy for younger patients with acute myeloid leukemia: results of the medical research council AML15 trial. J Clin Oncol. 2013;31:3360-8.

46. Burstein HJ, Cirrincione CT, Barry WT, Chew HK, Tolaney SM, Lake DE, et al. Endocrine therapy with or without inhibition of epidermal growth factor receptor and human epidermal growth factor receptor 2: a randomized, double-blind, place-bo-controlled phase III trial of fulvestrant with or without lapatinib for postmenopausal women with hormone receptor-positive advanced breast cancer-CALGB 40302 (Alliance). J Clin Oncol. 2014;32:3959-66.

47. Butts C, Socinski MA, Mitchell PL, Thatcher N, Havel L, Krzakowski M, et al. Tecemotide (L-BLP25) versus placebo after chemoradiotherapy for stage III non-small-cell lung cancer (START): a randomised, double-blind, phase 3 trial. Lancet Oncol. 2014;15:59-68.

48. Buzdar AU, Suman VJ, Meric-Bernstam F, Leitch AM, Ellis MJ, Boughey JC, et al. Fluorouracil, epirubicin, and cyclophosphamide (FEC-75) followed by paclitaxel plus trastuzumab versus paclitaxel plus trastuzumab followed by FEC-75 plus trastuzumab as neoadjuvant treatment for patients with HER2-positive breast cancer (Z1041): a random-ised, controlled, phase 3 trial. Lancet Oncol. 2013;14:1317-25.

49. Byrd JC, Brown JR, O'Brien S, Barrientos JC, Kay NE, Reddy NM, et al. Ibrutinib versus ofatumumab in previously treated chronic lymphoid leukemia. N Engl J Med. 2014;371:213-23.

50. Cainap C, Qin S, Huang WT, Chung IJ, Pan H, Cheng Y, et al. Linifanib versus Sorafenib in patients with advanced hepatocellular carcinoma: results of a randomized phase III trial. J Clin Oncol. 2015;33:172-9.

51. Cameron D, Brown J, Dent R, Jackisch C, Mackey J, Pivot X, et al. Adjuvant bevacizumab-containing therapy in triple-negative breast cancer (BEATRICE): primary results of a randomised, phase 3 trial. Lancet Oncol. 2013;14:933-42.

52. Caplin ME, Pavel M, Cwikla JB, Phan AT, Raderer M, Sedlackova E, et al. Lanreotide in metastatic enteropancreatic neuroendocrine tumors. N Engl J Med. 2014;371:224-33.

53. Carlson LE, Tamagawa R, Stephen J, Drysdale E, Zhong L, Speca M. Random-ized-controlled trial of mindfulness-based cancer recovery versus supportive expressive group therapy among distressed breast cancer survivors (MINDSET): long-term follow-up results. Psychooncology. 2016;25:750-9.

54. Carrato A, Swieboda-Sadlej A, Staszewska-Skurczynska M, Lim R, Roman L, Shparyk Y, et al. Fluorouracil, leucovorin, and irinotecan plus either sunitinib or placebo in metastatic colorectal cancer: a randomized, phase III trial. J Clin Oncol. 2013;31:1341-7.

55. Chagpar AB, Killelea BK, Tsangaris TN, Butler M, Stavris K, Li F, et al. A Ran-domized, Controlled Trial of Cavity Shave Margins in Breast Cancer. N Engl J Med. 2015;373:503-10.

56. Chanan-Khan A, Cramer P, Demirkan F, Fraser G, Silva RS, Grosicki S, et al. Ibrutinib combined with bendamustine and rituximab compared with placebo, benda-mustine, and rituximab for previously treated chronic lymphocytic leukaemia or small lymphocytic lymphoma (HELIOS): a randomised, double-blind, phase 3 study. Lancet Oncol. 2016;17:200-11.

57. Chandwani KD, Perkins G, Nagendra HR, Raghuram NV, Spelman A, Na-garathna R, et al. Randomized, controlled trial of yoga in women with breast cancer un-dergoing radiotherapy. J Clin Oncol. 2014;32:1058-65.

58. Cheng AL, Kang YK, Lin DY, Park JW, Kudo M, Qin S, et al. Sunitinib versus sorafenib in advanced hepatocellular cancer: results of a randomized phase III trial. J Clin Oncol. 2013;31:4067-75.

59. Chinot OL, Wick W, Mason W, Henriksson R, Saran F, Nishikawa R, et al. Bevacizumab plus radiotherapy-temozolomide for newly diagnosed glioblastoma. N Engl J Med. 2014;370:709-22.

60. Choueiri TK, Escudier B, Powles T, Mainwaring PN, Rini BI, Donskov F, et al. Cabozantinib versus Everolimus in Advanced Renal-Cell Carcinoma. N Engl J Med. 2015;373:1814-23.

61. Chow E, Meyer RM, Ding K, Nabid A, Chabot P, Wong P, et al. Dexamethasone in the prophylaxis of radiation-induced pain flare after palliative radiotherapy for bone metastases: a double-blind, randomised placebo-controlled, phase 3 trial. Lancet Oncol. 2015;16:1463-72.

62. Chow E, van der Linden YM, Roos D, Hartsell WF, Hoskin P, Wu JS, et al. Sin-gle versus multiple fractions of repeat radiation for painful bone metastases: a random-ised, controlled, non-inferiority trial. Lancet Oncol. 2014;15:164-71.

63. Cohen EE, Karrison TG, Kocherginsky M, Mueller J, Egan R, Huang CH, et al. Phase III randomized trial of induction chemotherapy in patients with N2 or N3 locally advanced head and neck cancer. J Clin Oncol. 2014;32:2735-43.

64. Coleman R, Cameron D, Dodwell D, Bell R, Wilson C, Rathbone E, et al. Adju-vant zoledronic acid in patients with early breast cancer: final efficacy analysis of the AZURE (BIG 01/04) randomised open-label phase 3 trial. Lancet Oncol. 2014;15:997-1006.

65. Conklin HM, Ogg RJ, Ashford JM, Scoggins MA, Zou P, Clark KN, et al. Com-puterized Cognitive Training for Amelioration of Cognitive Late Effects Among Childhood Cancer Survivors: A Randomized Controlled Trial. J Clin Oncol. 2015;33:3894-902.

66. Conroy T, Galais MP, Raoul JL, Bouche O, Gourgou-Bourgade S, Douillard JY, et al. Definitive chemoradiotherapy with FOLFOX versus fluorouracil and cisplatin in pa-tients with oesophageal cancer (PRODIGE5/ACCORD17): final results of a randomised, phase 2/3 trial. Lancet Oncol. 2014;15:305-14.

67. Cook G, Williams C, Brown JM, Cairns DA, Cavenagh J, Snowden JA, et al. High-dose chemotherapy plus autologous stem-cell transplantation as consolidation therapy in patients with relapsed multiple myeloma after previous autologous stem-cell transplantation (NCRI Myeloma X Relapse [Intensive trial]): a randomised, open-label, phase 3 trial. Lancet Oncol. 2014;15:874-85.

68. Cortes J, Dieras V, Ro J, Barriere J, Bachelot T, Hurvitz S, et al. Afatinib alone or afatinib plus vinorelbine versus investigator's choice of treatment for HER2-positive breast cancer with progressive brain metastases after trastuzumab, lapatinib, or both (LUX-Breast 3): a randomised, open-label, multicentre, phase 2 trial. Lancet Oncol. 2015;16:1700-10.

69. Cortes J, Feldman E, Yee K, Rizzieri D, Advani AS, Charman A, et al. Two dos-ing regimens of tosedostat in elderly patients with relapsed or refractory acute myeloid leukaemia (OPAL): a randomised open-label phase 2 study. Lancet Oncol. 2013;14:354-62.

70. Coudert B, Pierga JY, Mouret-Reynier MA, Kerrou K, Ferrero JM, Petit T, et al. Use of [(18)F]-FDG PET to predict response to neoadjuvant trastuzumab and docetaxel in patients with HER2-positive breast cancer, and addition of bevacizumab to neoadjuvant trastuzumab and docetaxel in [(18)F]-FDG PET-predicted non-responders (AVATAXHER): an open-label, randomised phase 2 trial. Lancet Oncol. 2014;15:1493-502.

71. Courneya KS, McKenzie DC, Mackey JR, Gelmon K, Friedenreich CM, Yasui Y, et al. Effects of exercise dose and type during breast cancer chemotherapy: multicenter randomized trial. J Natl Cancer Inst. 2013;105:1821-32.

72. Crosby T, Hurt CN, Falk S, Gollins S, Mukherjee S, Staffurth J, et al. Chemoradi-otherapy with or without cetuximab in patients with oesophageal cancer (SCOPE1): a multicentre, phase 2/3 randomised trial. Lancet Oncol. 2013;14:627-37.

73. Crown JP, Dieras V, Staroslawska E, Yardley DA, Bachelot T, Davidson N, et al. Phase III trial of sunitinib in combination with capecitabine versus capecitabine mono-therapy for the treatment of patients with pretreated metastatic breast cancer. J Clin Oncol. 2013;31:2870-8.

74. Crump M, Kuruvilla J, Couban S, MacDonald DA, Kukreti V, Kouroukis CT, et al. Randomized comparison of gemcitabine, dexamethasone, and cisplatin versus dexa-methasone, cytarabine, and cisplatin chemotherapy before autologous stem-cell trans-plantation for relapsed and refractory aggressive lymphomas: NCIC-CTG LY.12. J Clin Oncol. 2014;32:3490-6.

75. Cunningham D, Lang I, Marcuello E, Lorusso V, Ocvirk J, Shin DB, et al. Bevacizumab plus capecitabine versus capecitabine alone in elderly patients with previ-ously untreated metastatic colorectal cancer (AVEX): an open-label, randomised phase 3 trial. Lancet Oncol. 2013;14:1077-85.

76. D'Cruz AK, Vaish R, Kapre N, Dandekar M, Gupta S, Hawaldar R, et al. Elec-tive versus Therapeutic Neck Dissection in Node-Negative Oral Cancer. N Engl J Med. 2015;373:521-9.

77. Dayes IS, Whelan TJ, Julian JA, Parpia S, Pritchard KI, D'Souza DP, et al. Ran-domized trial of decongestive lymphatic therapy for the treatment of lymphedema in women with breast cancer. J Clin Oncol. 2013;31:3758-63.

78. de Raaf PJ, de Klerk C, Timman R, Busschbach JJ, Oldenmenger WH, van der Rijt CC. Systematic monitoring and treatment of physical symptoms to alleviate fatigue in patients with advanced cancer: a randomized controlled trial. J Clin Oncol. 2013;31:716-23.

79. Del Fabbro E, Dev R, Hui D, Palmer L, Bruera E. Effects of melatonin on appe-tite and other symptoms in patients with advanced cancer and cachexia: a double-blind placebo-controlled trial. J Clin Oncol. 2013;31:1271-6.

80. Delarue R, Tilly H, Mounier N, Petrella T, Salles G, Thieblemont C, et al. Dose-dense rituximab-CHOP compared with standard rituximab-CHOP in elderly pa-tients with diffuse large B-cell lymphoma (the LNH03-6B study): a randomised phase 3 trial. Lancet Oncol. 2013;14:525-33.

81. Demetri GD, Chawla SP, Ray-Coquard I, Le Cesne A, Staddon AP, Milhem MM, et al. Results of an international randomized phase III trial of the mammalian target of rapamycin inhibitor ridaforolimus versus placebo to control metastatic sarcomas in pa-tients after benefit from prior chemotherapy. J Clin Oncol. 2013;31:2485-92.

82. Denham JW, Joseph D, Lamb DS, Spry NA, Duchesne G, Matthews J, et al. Short-term androgen suppression and radiotherapy versus intermediate-term androgen suppression and radiotherapy, with or without zoledronic acid, in men with locally ad-vanced prostate cancer (TROG 03.04 RADAR): an open-label, randomised, phase 3 facto-rial trial. Lancet Oncol. 2014;15:1076-89.

83. Dimopoulos M, Siegel DS, Lonial S, Qi J, Hajek R, Facon T, et al. Vorinostat or placebo in combination with bortezomib in patients with multiple myeloma (VANTAGE 088): a multicentre, randomised, double-blind study. Lancet Oncol. 2013;14:1129-40.

84. DiSilvestro PA, Ali S, Craighead PS, Lucci JA, Lee YC, Cohn DE, et al. Phase III randomized trial of weekly cisplatin and irradiation versus cisplatin and tirapazamine and irradiation in stages IB2, IIA, IIB, IIIB, and IVA cervical carcinoma limited to the pel-vis: a Gynecologic Oncology Group study. J Clin Oncol. 2014;32:458-64.

85. Dobs AS, Boccia RV, Croot CC, Gabrail NY, Dalton JT, Hancock ML, et al. Ef-fects of enobosarm on muscle wasting and physical function in patients with cancer: a double-blind, randomised controlled phase 2 trial. Lancet Oncol. 2013;14:335-45.

86. Donker M, van Tienhoven G, Straver ME, Meijnen P, van de Velde CJ, Mansel RE, et al. Radiotherapy or surgery of the axilla after a positive sentinel node in breast can-cer (EORTC 10981-22023 AMAROS): a randomised, multicentre, open-label, phase 3 non-inferiority trial. Lancet Oncol. 2014;15:1303-10.

87. du Bois A, Floquet A, Kim JW, Rau J, del Campo JM, Friedlander M, et al. In-corporation of pazopanib in maintenance therapy of ovarian cancer. J Clin Oncol. 2014;32:3374-82.

88. Dutton SJ, Ferry DR, Blazeby JM, Abbas H, Dahle-Smith A, Mansoor W, et al. Gefitinib for oesophageal cancer progressing after chemotherapy (COG): a phase 3, multi-centre, double-blind, placebo-controlled randomised trial. Lancet Oncol. 2014;15:894-904.

89. Earl HM, Hiller L, Dunn JA, Blenkinsop C, Grybowicz L, Vallier AL, et al. Effi-cacy of neoadjuvant bevacizumab added to docetaxel followed by fluorouracil, epirubicin, and cyclophosphamide, for women with HER2-negative early breast cancer (ARTemis): an open-label, randomised, phase 3 trial. Lancet Oncol. 2015;16:656-66.

90. Earl HM, Vallier AL, Hiller L, Fenwick N, Young J, Iddawela M, et al. Effects of the addition of gemcitabine, and paclitaxel-first sequencing, in neoadjuvant sequential epirubicin, cyclophosphamide, and paclitaxel for women with high-risk early breast can-cer (Neo-tAnGo): an open-label, 2x2 factorial randomised phase 3 trial. Lancet Oncol. 2014;15:201-12.

91. Eberhardt WE, Pottgen C, Gauler TC, Friedel G, Veit S, Heinrich V, et al. Phase III Study of Surgery Versus Definitive Concurrent Chemoradiotherapy Boost in Patients With Resectable Stage IIIA(N2) and Selected IIIB Non-Small-Cell Lung Cancer After Induc-tion Chemotherapy and Concurrent Chemoradiotherapy (ESPATUE). J Clin Oncol. 2015;33:4194-201.

92. Edelman MJ, Schneider CP, Tsai CM, Kim HT, Quoix E, Luft AV, et al. Ran-domized phase II study of ixabepilone or paclitaxel plus carboplatin in patients with non-small-cell lung cancer prospectively stratified by beta-3 tubulin status. J Clin Oncol. 2013;31:1990-6.

93. Edelman MJ, Tan MT, Fidler MJ, Sanborn RE, Otterson G, Sequist LV, et al. Randomized, double-blind, placebo-controlled, multicenter phase II study of the efficacy and safety of apricoxib in combination with either docetaxel or pemetrexed in patients with biomarker-selected non-small-cell lung cancer. J Clin Oncol. 2015;33:189-94.

94. Eeles RA, Morden JP, Gore M, Mansi J, Glees J, Wenczl M, et al. Adjuvant Hor-mone Therapy May Improve Survival in Epithelial Ovarian Cancer: Results of the AHT Randomized Trial. J Clin Oncol. 2015;33:4138-44.

95. Eggermont AM, Chiarion-Sileni V, Grob JJ, Dummer R, Wolchok JD, Schmidt H, et al. Adjuvant ipilimumab versus placebo after complete resection of high-risk stage III melanoma (EORTC 18071): a randomised, double-blind, phase 3 trial. Lancet Oncol. 2015;16:522-30.

96. Eggermont AM, Suciu S, Rutkowski P, Marsden J, Santinami M, Corrie P, et al. Adjuvant ganglioside GM2-KLH/QS-21 vaccination versus observation after resection of primary tumor > 1.5 mm in patients with stage II melanoma: results of the EORTC 18961 randomized phase III trial. J Clin Oncol. 2013;31:3831-7.

97. Elisei R, Schlumberger MJ, Muller SP, Schoffski P, Brose MS, Shah MH, et al. Cabozantinib in progressive medullary thyroid cancer. J Clin Oncol. 2013;31:3639-46.

98. Ellis PM, Shepherd FA, Millward M, Perrone F, Seymour L, Liu G, et al. Dacomitinib compared with placebo in pretreated patients with advanced or metastatic non-small-cell lung cancer (NCIC CTG BR.26): a double-blind, randomised, phase 3 trial. Lancet Oncol. 2014;15:1379-88.

99. Escudier B, Porta C, Bono P, Powles T, Eisen T, Sternberg CN, et al. Random-ized, controlled, double-blind, cross-over trial assessing treatment preference for pazo-panib versus sunitinib in patients with metastatic renal cell carcinoma: PISCES Study. J Clin Oncol. 2014;32:1412-8.

100. Fassnacht M, Berruti A, Baudin E, Demeure MJ, Gilbert J, Haak H, et al. Linsit-inib (OSI-906) versus placebo for patients with locally advanced or metastatic adrenocor-tical carcinoma: a double-blind, randomised, phase 3 study. Lancet Oncol. 2015;16:426-35.

101. Federico M, Luminari S, Dondi A, Tucci A, Vitolo U, Rigacci L, et al. R-CVP versus R-CHOP versus R-FM for the initial treatment of patients with advanced-stage fol-licular lymphoma: results of the FOLL05 trial conducted by the Fondazione Italiana Lin-fomi. J Clin Oncol. 2013;31:1506-13.

102. Fernando HC, Landreneau RJ, Mandrekar SJ, Nichols FC, Hillman SL, Heron DE, et al. Impact of brachytherapy on local recurrence rates after sublobar resection: results from ACOSOG Z4032 (Alliance), a phase III randomized trial for high-risk operable non-small-cell lung cancer. J Clin Oncol. 2014;32:2456-62.

103. Finn RS, Crown JP, Lang I, Boer K, Bondarenko IM, Kulyk SO, et al. The cy-clin-dependent kinase 4/6 inhibitor palbociclib in combination with letrozole versus let-rozole alone as first-line treatment of oestrogen receptor-positive, HER2-negative, ad-vanced breast cancer (PALOMA-1/TRIO-18): a randomised phase 2 study. Lancet Oncol. 2015;16:25-35.

104. Fizazi K, Faivre L, Lesaunier F, Delva R, Gravis G, Rolland F, et al. Androgen deprivation therapy plus docetaxel and estramustine versus androgen deprivation thera-py alone for high-risk localised prostate cancer (GETUG 12): a phase 3 randomised con-trolled trial. Lancet Oncol. 2015;16:787-94.

105. Fizazi K, Higano CS, Nelson JB, Gleave M, Miller K, Morris T, et al. Phase III, randomized, placebo-controlled study of docetaxel in combination with zibotentan in pa-tients with metastatic castration-resistant prostate cancer. J Clin Oncol. 2013;31:1740-7.

106. Fizazi K, Jones R, Oudard S, Efstathiou E, Saad F, de Wit R, et al. Phase III, ran-domized, double-blind, multicenter trial comparing orteronel (TAK-700) plus prednisone with placebo plus prednisone in patients with metastatic castration-resistant prostate cancer that has progressed during or after docetaxel-based therapy: ELM-PC 5. J Clin On-col. 2015;33:723-31.

107. Fizazi K, Pagliaro L, Laplanche A, Flechon A, Mardiak J, Geoffrois L, et al. Per-sonalised chemotherapy based on tumour marker decline in poor prognosis germ-cell tumours (GETUG 13): a phase 3, multicentre, randomised trial. Lancet Oncol. 2014;15:1442-50.

108. Flaherty KT, Lee SJ, Zhao F, Schuchter LM, Flaherty L, Kefford R, et al. Phase III trial of carboplatin and paclitaxel with or without sorafenib in metastatic melanoma. J Clin Oncol. 2013;31:373-9.

109. Flaherty KT, Manola JB, Pins M, McDermott DF, Atkins MB, Dutcher JJ, et al. BEST: A Randomized Phase II Study of Vascular Endothelial Growth Factor, RAF Kinase, and Mammalian Target of Rapamycin Combination Targeted Therapy With Bevaci-zumab, Sorafenib, and Temsirolimus in Advanced Renal Cell Carcinoma--A Trial of the ECOG-ACRIN Cancer Research Group (E2804). J Clin Oncol. 2015;33:2384-91.

110. Flaherty LE, Othus M, Atkins MB, Tuthill RJ, Thompson JA, Vetto JT, et al. Southwest Oncology Group S0008: a phase III trial of high-dose interferon Alfa-2b versus cisplatin, vinblastine, and dacarbazine, plus interleukin-2 and interferon in patients with high-risk melanoma--an intergroup study of cancer and leukemia Group B, Children's Oncology Group, Eastern Cooperative Oncology Group, and Southwest Oncology Group. J Clin Oncol. 2014;32:3771-8.

111. Ford HE, Marshall A, Bridgewater JA, Janowitz T, Coxon FY, Wadsley J, et al. Docetaxel versus active symptom control for refractory oesophagogastric adenocarcinoma (COUGAR-02): an open-label, phase 3 randomised controlled trial. Lancet Oncol. 2014;15:78-86.

112. Francis PA, Regan MM, Fleming GF, Lang I, Ciruelos E, Bellet M, et al. Adju-vant ovarian suppression in premenopausal breast cancer. N Engl J Med. 2015;372:436-46.

113. Friedman DL, Chen L, Wolden S, Buxton A, McCarten K, FitzGerald TJ, et al. Dose-intensive response-based chemotherapy and radiation therapy for children and ad-olescents with newly diagnosed intermediate-risk hodgkin lymphoma: a report from the Children's Oncology Group Study AHOD0031. J Clin Oncol. 2014;32:3651-8.

114. Furman RR, Sharman JP, Coutre SE, Cheson BD, Pagel JM, Hillmen P, et al. Idelalisib and rituximab in relapsed chronic lymphocytic leukemia. N Engl J Med. 2014;370:997-1007.

115. Galimberti V, Cole BF, Zurrida S, Viale G, Luini A, Veronesi P, et al. Axillary dissection versus no axillary dissection in patients with sentinel-node micrometastases (IBCSG 23-01): a phase 3 randomised controlled trial. Lancet Oncol. 2013;14:297-305.

116. Gamis AS, Alonzo TA, Meshinchi S, Sung L, Gerbing RB, Raimondi SC, et al. Gemtuzumab ozogamicin in children and adolescents with de novo acute myeloid leu-kemia improves event-free survival by reducing relapse risk: results from the randomized phase III Children's Oncology Group trial AAML0531. J Clin Oncol. 2014;32:3021-32.

117. Garassino MC, Martelli O, Broggini M, Farina G, Veronese S, Rulli E, et al. Erlo-tinib versus docetaxel as second-line treatment of patients with advanced non-small-cell lung cancer and wild-type EGFR tumours (TAILOR): a randomised controlled trial. Lancet Oncol. 2013;14:981-8.

118. Garcia-Manero G, Jabbour E, Borthakur G, Faderl S, Estrov Z, Yang H, et al. Randomized open-label phase II study of decitabine in patients with low- or intermedi-ate-risk myelodysplastic syndromes. J Clin Oncol. 2013;31:2548-53.

119. Garland SN, Carlson LE, Stephens AJ, Antle MC, Samuels C, Campbell TS. Mindfulness-based stress reduction compared with cognitive behavioral therapy for the treatment of insomnia comorbid with cancer: a randomized, partially blinded, noninferi-ority trial. J Clin Oncol. 2014;32:449-57.

120. Gay F, Oliva S, Petrucci MT, Conticello C, Catalano L, Corradini P, et al. Chem-otherapy plus lenalidomide versus autologous transplantation, followed by lenalidomide plus prednisone versus lenalidomide maintenance, in patients with multiple myeloma: a randomised, multicentre, phase 3 trial. Lancet Oncol. 2015;16:1617-29.

121. Gelmon KA, Boyle FM, Kaufman B, Huntsman DG, Manikhas A, Di Leo A, et al. Lapatinib or Trastuzumab Plus Taxane Therapy for Human Epidermal Growth Factor Receptor 2-Positive Advanced Breast Cancer: Final Results of NCIC CTG MA.31. J Clin Oncol. 2015;33:1574-83.

122. Ghadjar P, Hayoz S, Bernhard J, Zwahlen DR, Holscher T, Gut P, et al. Acute Toxicity and Quality of Life After Dose-Intensified Salvage Radiation Therapy for Bio-chemically Recurrent Prostate Cancer After Prostatectomy: First Results of the Random-ized Trial SAKK 09/10. J Clin Oncol. 2015;33:4158-66.

123. Gianni L, Romieu GH, Lichinitser M, Serrano SV, Mansutti M, Pivot X, et al. AVEREL: a randomized phase III Trial evaluating bevacizumab in combination with docetaxel and trastuzumab as first-line therapy for HER2-positive locally recur-rent/metastatic breast cancer. J Clin Oncol. 2013;31:1719-25.

124. Gilbert MR, Dignam JJ, Armstrong TS, Wefel JS, Blumenthal DT, Vogelbaum MA, et al. A randomized trial of bevacizumab for newly diagnosed glioblastoma. N Engl J Med. 2014;370:699-708.

125. Gilbert MR, Wang M, Aldape KD, Stupp R, Hegi ME, Jaeckle KA, et al. Dose-dense temozolomide for newly diagnosed glioblastoma: a randomized phase III clinical trial. J Clin Oncol. 2013;31:4085-91.

126. Giralt J, Trigo J, Nuyts S, Ozsahin M, Skladowski K, Hatoum G, et al. Pani-tumumab plus radiotherapy versus chemoradiotherapy in patients with unresected, lo-cally advanced squamous-cell carcinoma of the head and neck (CONCERT-2): a random-ised, controlled, open-label phase 2 trial. Lancet Oncol. 2015;16:221-32.

127. Glass B, Hasenkamp J, Wulf G, Dreger P, Pfreundschuh M, Gramatzki M, et al. Rituximab after lymphoma-directed conditioning and allogeneic stem-cell transplanta-tion for relapsed and refractory aggressive non-Hodgkin lymphoma (DSHNHL R3): an open-label, randomised, phase 2 trial. Lancet Oncol. 2014;15:757-66.

128. Gligorov J, Doval D, Bines J, Alba E, Cortes P, Pierga JY, et al. Maintenance capecitabine and bevacizumab versus bevacizumab alone after initial first-line bevaci-zumab and docetaxel for patients with HER2-negative metastatic breast cancer (IMELDA): a randomised, open-label, phase 3 trial. Lancet Oncol. 2014;15:1351-60.

129. Glover M, Smerdon GR, Andreyev HJ, Benton BE, Bothma P, Firth O, et al. Hy-perbaric oxygen for patients with chronic bowel dysfunction after pelvic radiotherapy (HOT2): a randomised, double-blind, sham-controlled phase 3 trial. Lancet Oncol. 2016;17:224-33.

130. Goede V, Fischer K, Busch R, Engelke A, Eichhorst B, Wendtner CM, et al. Obinutuzumab plus chlorambucil in patients with CLL and coexisting conditions. N Engl J Med. 2014;370:1101-10.

131. Goetsch MF, Lim JY, Caughey AB. A Practical Solution for Dyspareunia in Breast Cancer Survivors: A Randomized Controlled Trial. J Clin Oncol. 2015;33:3394-400.

132. Goodwin PJ, Segal RJ, Vallis M, Ligibel JA, Pond GR, Robidoux A, et al. Ran-domized trial of a telephone-based weight loss intervention in postmenopausal women with breast cancer receiving letrozole: the LISA trial. J Clin Oncol. 2014;32:2231-9.

133. Gordon LI, Hong F, Fisher RI, Bartlett NL, Connors JM, Gascoyne RD, et al. Randomized phase III trial of ABVD versus Stanford V with or without radiation therapy in locally extensive and advanced-stage Hodgkin lymphoma: an intergroup study coor-dinated by the Eastern Cooperative Oncology Group (E2496). J Clin Oncol. 2013;31:684-91.

134. Goss GD, O'Callaghan C, Lorimer I, Tsao MS, Masters GA, Jett J, et al. Gefitinib versus placebo in completely resected non-small-cell lung cancer: results of the NCIC CTG BR19 study. J Clin Oncol. 2013;31:3320-6.

135. Goss PE, Ingle JN, Pritchard KI, Ellis MJ, Sledge GW, Budd GT, et al. Exemes-tane versus anastrozole in postmenopausal women with early breast cancer: NCIC CTG MA.27--a randomized controlled phase III trial. J Clin Oncol. 2013;31:1398-404.

136. Goss PE, Smith IE, O'Shaughnessy J, Ejlertsen B, Kaufmann M, Boyle F, et al. Adjuvant lapatinib for women with early-stage HER2-positive breast cancer: a random-ised, controlled, phase 3 trial. Lancet Oncol. 2013;14:88-96.

137. Gravis G, Fizazi K, Joly F, Oudard S, Priou F, Esterni B, et al. Andro-gen-deprivation therapy alone or with docetaxel in non-castrate metastatic prostate cancer (GETUG-AFU 15): a randomised, open-label, phase 3 trial. Lancet Oncol. 2013;14:149-58.

138. Gregorc V, Novello S, Lazzari C, Barni S, Aieta M, Mencoboni M, et al. Predic-tive value of a proteomic signature in patients with non-small-cell lung cancer treated with second-line erlotinib or chemotherapy (PROSE): a biomarker-stratified, randomised phase 3 trial. Lancet Oncol. 2014;15:713-21.

139. Guan Z, Xu B, DeSilvio ML, Shen Z, Arpornwirat W, Tong Z, et al. Randomized trial of lapatinib versus placebo added to paclitaxel in the treatment of human epidermal growth factor receptor 2-overexpressing metastatic breast cancer. J Clin Oncol. 2013;31:1947-53.

140. Guarneri V, Generali DG, Frassoldati A, Artioli F, Boni C, Cavanna L, et al. Double-blind, placebo-controlled, multicenter, randomized, phase IIb neoadjuvant study of letrozole-lapatinib in postmenopausal hormone receptor-positive, human epidermal growth factor receptor 2-negative, operable breast cancer. J Clin Oncol. 2014;32:1050-7.

141. Guimbaud R, Louvet C, Ries P, Ychou M, Maillard E, Andre T, et al. Prospec-tive, randomized, multicenter, phase III study of fluorouracil, leucovorin, and irinotecan versus epirubicin, cisplatin, and capecitabine in advanced gastric adenocarcinoma: a French intergroup (Federation Francophone de Cancerologie Digestive, Federation Natio-nale des Centres de Lutte Contre le Cancer, and Groupe Cooperateur Multidisciplinaire en Oncologie) study. J Clin Oncol. 2014;32:3520-6.

142. Haddad R, O'Neill A, Rabinowits G, Tishler R, Khuri F, Adkins D, et al. Induc-tion chemotherapy followed by concurrent chemoradiotherapy (sequential chemoradio-therapy) versus concurrent chemoradiotherapy alone in locally advanced head and neck cancer (PARADIGM): a randomised phase 3 trial. Lancet Oncol. 2013;14:257-64.

143. Harari PM, Harris J, Kies MS, Myers JN, Jordan RC, Gillison ML, et al. Postop-erative chemoradiotherapy and cetuximab for high-risk squamous cell carcinoma of the head and neck: Radiation Therapy Oncology Group RTOG-0234. J Clin Oncol. 2014;32:2486-95.

144. Harrington K, Temam S, Mehanna H, D'Cruz A, Jain M, D'Onofrio I, et al. Postoperative Adjuvant Lapatinib and Concurrent Chemoradiotherapy Followed by Maintenance Lapatinib Monotherapy in High-Risk Patients With Resected Squamous Cell Carcinoma of the Head and Neck: A Phase III, Randomized, Double-Blind, Place-bo-Controlled Study. J Clin Oncol. 2015;33:4202-9.

145. Heinemann V, von Weikersthal LF, Decker T, Kiani A, Vehling-Kaiser U, Al-Batran SE, et al. FOLFIRI plus cetuximab versus FOLFIRI plus bevacizumab as first-line treatment for patients with metastatic colorectal cancer (FIRE-3): a randomised, open-label, phase 3 trial. Lancet Oncol. 2014;15:1065-75.

146. Hensley ML, Miller A, O'Malley DM, Mannel RS, Behbakht K, Bakkum-Gamez JN, et al. Randomized phase III trial of gemcitabine plus docetaxel plus bevacizumab or placebo as first-line treatment for metastatic uterine leiomyosarcoma: an NRG Oncolo-gy/Gynecologic Oncology Group study. J Clin Oncol. 2015;33:1180-5.

147. Herman JM, Wild AT, Wang H, Tran PT, Chang KJ, Taylor GE, et al. Random-ized phase III multi-institutional study of TNFerade biologic with fluorouracil and radio-therapy for locally advanced pancreatic cancer: final results. J Clin Oncol. 2013;31:886-94.

148. Hershman DL, Unger JM, Crew KD, Awad D, Dakhil SR, Gralow J, et al. Ran-domized Multicenter Placebo-Controlled Trial of Omega-3 Fatty Acids for the Control of Aromatase Inhibitor-Induced Musculoskeletal Pain: SWOG S0927. J Clin Oncol. 2015;33:1910-7.

149. Hershman DL, Unger JM, Crew KD, Minasian LM, Awad D, Moinpour CM, et al. Randomized double-blind placebo-controlled trial of acetyl-L-carnitine for the preven-tion of taxane-induced neuropathy in women undergoing adjuvant breast cancer therapy. J Clin Oncol. 2013;31:2627-33.

150. Hironaka S, Ueda S, Yasui H, Nishina T, Tsuda M, Tsumura T, et al. Random-ized, open-label, phase III study comparing irinotecan with paclitaxel in patients with advanced gastric cancer without severe peritoneal metastasis after failure of prior combi-nation chemotherapy using fluoropyrimidine plus platinum: WJOG 4007 trial. J Clin On-col. 2013;31:4438-44.

151. Hoff PM, Saragiotto DF, Barrios CH, del Giglio A, Coutinho AK, Andrade AC, et al. Randomized phase III trial exploring the use of long-acting release octreotide in the prevention of chemotherapy-induced diarrhea in patients with colorectal cancer: the LARCID trial. J Clin Oncol. 2014;32:1006-11.

152. Hofheinz RD, Gencer D, Schulz H, Stahl M, Hegewisch-Becker S, Loeffler LM, et al. Mapisal Versus Urea Cream as Prophylaxis for Capecitabine-Associated Hand-Foot Syndrome: A Randomized Phase III Trial of the AIO Quality of Life Working Group. J Clin Oncol. 2015;33:2444-9.

153. Hong YS, Nam BH, Kim KP, Kim JE, Park SJ, Park YS, et al. Oxaliplatin, fluor-ouracil, and leucovorin versus fluorouracil and leucovorin as adjuvant chemotherapy for locally advanced rectal cancer after preoperative chemoradiotherapy (ADORE): an open-label, multicentre, phase 2, randomised controlled trial. Lancet Oncol. 2014;15:1245-53.

154. Hoskin P, Sundar S, Reczko K, Forsyth S, Mithal N, Sizer B, et al. A Multicenter Randomized Trial of Ibandronate Compared With Single-Dose Radiotherapy for Local-ized Metastatic Bone Pain in Prostate Cancer. J Natl Cancer Inst. 2015;107.

155. Hoskin PJ, Kirkwood AA, Popova B, Smith P, Robinson M, Gallop-Evans E, et al. 4 Gy versus 24 Gy radiotherapy for patients with indolent lymphoma (FORT): a ran-domised phase 3 non-inferiority trial. Lancet Oncol. 2014;15:457-63.

156. Hu XC, Zhang J, Xu BH, Cai L, Ragaz J, Wang ZH, et al. Cisplatin plus gem-citabine versus paclitaxel plus gemcitabine as first-line therapy for metastatic tri-ple-negative breast cancer (CBCSG006): a randomised, open-label, multicentre, phase 3 trial. Lancet Oncol. 2015;16:436-46.

157. Huang YH, Hsiao LT, Hong YC, Chiou TJ, Yu YB, Gau JP, et al. Randomized controlled trial of entecavir prophylaxis for rituximab-associated hepatitis B virus reacti-vation in patients with lymphoma and resolved hepatitis B. J Clin Oncol. 2013;31:2765-72.

158. Hurvitz SA, Andre F, Jiang Z, Shao Z, Mano MS, Neciosup SP, et al. Combina-tion of everolimus with trastuzumab plus paclitaxel as first-line treatment for patients with HER2-positive advanced breast cancer (BOLERO-1): a phase 3, randomised, dou-ble-blind, multicentre trial. Lancet Oncol. 2015;16:816-29.

159. Hurvitz SA, Dirix L, Kocsis J, Bianchi GV, Lu J, Vinholes J, et al. Phase II ran-domized study of trastuzumab emtansine versus trastuzumab plus docetaxel in patients with human epidermal growth factor receptor 2-positive metastatic breast cancer. J Clin Oncol. 2013;31:1157-63.

160. Hurwitz HI, Uppal N, Wagner SA, Bendell JC, Beck JT, Wade SM, 3rd, et al. Randomized, Double-Blind, Phase II Study of Ruxolitinib or Placebo in Combination With Capecitabine in Patients With Metastatic Pancreatic Cancer for Whom Therapy With Gemcitabine Has Failed. J Clin Oncol. 2015;33:4039-47.

161. Hurwitz MD, Ghanouni P, Kanaev SV, Iozeffi D, Gianfelice D, Fennessy FM, et al. Magnetic resonance-guided focused ultrasound for patients with painful bone metas-tases: phase III trial results. J Natl Cancer Inst. 2014;106.

162. Hussain M, Tangen CM, Berry DL, Higano CS, Crawford ED, Liu G, et al. In-termittent versus continuous androgen deprivation in prostate cancer. N Engl J Med. 2013;368:1314-25.

163. Hutson TE, Escudier B, Esteban E, Bjarnason GA, Lim HY, Pittman KB, et al. Randomized phase III trial of temsirolimus versus sorafenib as second-line therapy after sunitinib in patients with metastatic renal cell carcinoma. J Clin Oncol. 2014;32:760-7.

164. Hutson TE, Lesovoy V, Al-Shukri S, Stus VP, Lipatov ON, Bair AH, et al. Ax-itinib versus sorafenib as first-line therapy in patients with metastatic renal-cell carcino-ma: a randomised open-label phase 3 trial. Lancet Oncol. 2013;14:1287-94.

165. Irwin ML, Cartmel B, Gross CP, Ercolano E, Li F, Yao X, et al. Randomized ex-ercise trial of aromatase inhibitor-induced arthralgia in breast cancer survivors. J Clin Oncol. 2015;33:1104-11.

166. Ito A, Shintaku I, Satoh M, Ioritani N, Aizawa M, Tochigi T, et al. Prospective randomized phase II trial of a single early intravesical instillation of pirarubicin (THP) in the prevention of bladder recurrence after nephroureterectomy for upper urinary tract urothelial carcinoma: the THP Monotherapy Study Group Trial. J Clin Oncol. 2013;31:1422-7.

167. James RD, Glynne-Jones R, Meadows HM, Cunningham D, Myint AS, Saun-ders MP, et al. Mitomycin or cisplatin chemoradiation with or without maintenance chemotherapy for treatment of squamous-cell carcinoma of the anus (ACT II): a random-ised, phase 3, open-label, 2 x 2 factorial trial. Lancet Oncol. 2013;14:516-24.

168. Janne PA, Shaw AT, Pereira JR, Jeannin G, Vansteenkiste J, Barrios C, et al. Se-lumetinib plus docetaxel for KRAS-mutant advanced non-small-cell lung cancer: a ran-domised, multicentre, placebo-controlled, phase 2 study. Lancet Oncol. 2013;14:38-47.

169. Jeong SY, Park JW, Nam BH, Kim S, Kang SB, Lim SB, et al. Open versus lapa-roscopic surgery for mid-rectal or low-rectal cancer after neoadjuvant chemoradiotherapy (COREAN trial): survival outcomes of an open-label, non-inferiority, randomised con-trolled trial. Lancet Oncol. 2014;15:767-74.

170. Ji Y, Rankin C, Grunberg S, Sherrod AE, Ahmadi J, Townsend JJ, et al. Dou-ble-Blind Phase III Randomized Trial of the Antiprogestin Agent Mifepristone in the Treatment of Unresectable Meningioma: SWOG S9005. J Clin Oncol. 2015;33:4093-8.

171. Jin J, Wang JX, Chen FF, Wu DP, Hu J, Zhou JF, et al. Homoharringtonine-based induction regimens for patients with de-novo acute myeloid leukaemia: a multicentre, open-label, randomised, controlled phase 3 trial. Lancet Oncol. 2013;14:599-608.

172. Johnson BE, Kabbinavar F, Fehrenbacher L, Hainsworth J, Kasubhai S, Kressel B, et al. ATLAS: randomized, double-blind, placebo-controlled, phase IIIB trial comparing bevacizumab therapy with or without erlotinib, after completion of chemotherapy, with bevacizumab for first-line treatment of advanced non-small-cell lung cancer. J Clin Oncol. 2013;31:3926-34.

173. Johnson PJ, Qin S, Park JW, Poon RT, Raoul JL, Philip PA, et al. Brivanib versus sorafenib as first-line therapy in patients with unresectable, advanced hepatocellular car-cinoma: results from the randomized phase III BRISK-FL study. J Clin Oncol. 2013;31:3517-24.

174. Johnston SR, Kilburn LS, Ellis P, Dodwell D, Cameron D, Hayward L, et al. Fulvestrant plus anastrozole or placebo versus exemestane alone after progression on non-steroidal aromatase inhibitors in postmenopausal patients with hor-mone-receptor-positive locally advanced or metastatic breast cancer (SoFEA): a composite, multicentre, phase 3 randomised trial. Lancet Oncol. 2013;14:989-98.

175. Judson I, Verweij J, Gelderblom H, Hartmann JT, Schoffski P, Blay JY, et al. Doxorubicin alone versus intensified doxorubicin plus ifosfamide for first-line treatment of advanced or metastatic soft-tissue sarcoma: a randomised controlled phase 3 trial. Lancet Oncol. 2014;15:415-23.

176. Kahl BS, Hong F, Williams ME, Gascoyne RD, Wagner LI, Krauss JC, et al. Rituximab extended schedule or re-treatment trial for low-tumor burden follicular lym-phoma: eastern cooperative oncology group protocol e4402. J Clin Oncol. 2014;32:3096-102.

177. Kang HJ, Loftus S, Taylor A, DiCristina C, Green S, Zwaan CM. Aprepitant for the prevention of chemotherapy-induced nausea and vomiting in children: a randomised, double-blind, phase 3 trial. Lancet Oncol. 2015;16:385-94.

178. Kang YK, Ryu MH, Yoo C, Ryoo BY, Kim HJ, Lee JJ, et al. Resumption of imatinib to control metastatic or unresectable gastrointestinal stromal tumours after fail-ure of imatinib and sunitinib (RIGHT): a randomised, placebo-controlled, phase 3 trial. Lancet Oncol. 2013;14:1175-82.

179. Kaspers GJ, Zimmermann M, Reinhardt D, Gibson BE, Tamminga RY, Aleini-kova O, et al. Improved outcome in pediatric relapsed acute myeloid leukemia: results of a randomized trial on liposomal daunorubicin by the International BFM Study Group. J Clin Oncol. 2013;31:599-607.

180. Kaufman PA, Awada A, Twelves C, Yelle L, Perez EA, Velikova G, et al. Phase III open-label randomized study of eribulin mesylate versus capecitabine in patients with locally advanced or metastatic breast cancer previously treated with an anthracycline and a taxane. J Clin Oncol. 2015;33:594-601.

181. Kawaguchi T, Ando M, Asami K, Okano Y, Fukuda M, Nakagawa H, et al. Randomized phase III trial of erlotinib versus docetaxel as second- or third-line therapy in patients with advanced non-small-cell lung cancer: Docetaxel and Erlotinib Lung Cancer Trial (DELTA). J Clin Oncol. 2014;32:1902-8.

182. Kawai A, Araki N, Sugiura H, Ueda T, Yonemoto T, Takahashi M, et al. Tra-bectedin monotherapy after standard chemotherapy versus best supportive care in pa-tients with advanced, translocation-related sarcoma: a randomised, open-label, phase 2 study. Lancet Oncol. 2015;16:406-16.

183. Kellokumpu-Lehtinen PL, Harmenberg U, Joensuu T, McDermott R, Hervonen P, Ginman C, et al. 2-Weekly versus 3-weekly docetaxel to treat castration-resistant ad-vanced prostate cancer: a randomised, phase 3 trial. Lancet Oncol. 2013;14:117-24.

184. Kelly K, Altorki NK, Eberhardt WE, O'Brien ME, Spigel DR, Crino L, et al. Ad-juvant Erlotinib Versus Placebo in Patients With Stage IB-IIIA Non-Small-Cell Lung Can-cer (RADIANT): A Randomized, Double-Blind, Phase III Trial. J Clin Oncol. 2015;33:4007-14.

185. Kennedy RH, Francis EA, Wharton R, Blazeby JM, Quirke P, West NP, et al. Multicenter randomized controlled trial of conventional versus laparoscopic surgery for colorectal cancer within an enhanced recovery programme: EnROL. J Clin Oncol. 2014;32:1804-11.

186. Kern WV, Marchetti O, Drgona L, Akan H, Aoun M, Akova M, et al. Oral anti-biotics for fever in low-risk neutropenic patients with cancer: a double-blind, randomized, multicenter trial comparing single daily moxifloxacin with twice daily ciprofloxacin plus amoxicillin/clavulanic acid combination therapy--EORTC infectious diseases group trial XV. J Clin Oncol. 2013;31:1149-56.

187. Kiecolt-Glaser JK, Bennett JM, Andridge R, Peng J, Shapiro CL, Malarkey WB, et al. Yoga's impact on inflammation, mood, and fatigue in breast cancer survivors: a ran-domized controlled trial. J Clin Oncol. 2014;32:1040-9.

188. Kim ES, Neubauer M, Cohn A, Schwartzberg L, Garbo L, Caton J, et al. Docet-axel or pemetrexed with or without cetuximab in recurrent or progressive non-small-cell lung cancer after platinum-based therapy: a phase 3, open-label, randomised trial. Lancet Oncol. 2013;14:1326-36.

189. Kitagawa R, Katsumata N, Shibata T, Kamura T, Kasamatsu T, Nakanishi T, et al. Paclitaxel Plus Carboplatin Versus Paclitaxel Plus Cisplatin in Metastatic or Recurrent Cervical Cancer: The Open-Label Randomized Phase III Trial JCOG0505. J Clin Oncol. 2015;33:2129-35.

190. Kordes S, Pollak MN, Zwinderman AH, Mathot RA, Weterman MJ, Beeker A, et al. Metformin in patients with advanced pancreatic cancer: a double-blind, randomised, placebo-controlled phase 2 trial. Lancet Oncol. 2015;16:839-47.

191. Kreissman SG, Seeger RC, Matthay KK, London WB, Sposto R, Grupp SA, et al. Purged versus non-purged peripheral blood stem-cell transplantation for high-risk neuro-blastoma (COG A3973): a randomised phase 3 trial. Lancet Oncol. 2013;14:999-1008.

192. Krekel NM, Haloua MH, Lopes Cardozo AM, de Wit RH, Bosch AM, de Widt-Levert LM, et al. Intraoperative ultrasound guidance for palpable breast cancer exci-sion (COBALT trial): a multicentre, randomised controlled trial. Lancet Oncol. 2013;14:48-54.

193. Krop IE, Kim SB, Gonzalez-Martin A, LoRusso PM, Ferrero JM, Smitt M, et al. Trastuzumab emtansine versus treatment of physician's choice for pretreated HER2-positive advanced breast cancer (TH3RESA): a randomised, open-label, phase 3 tri-al. Lancet Oncol. 2014;15:689-99.

194. Krug LM, Kindler HL, Calvert H, Manegold C, Tsao AS, Fennell D, et al. Vori-nostat in patients with advanced malignant pleural mesothelioma who have progressed on previous chemotherapy (VANTAGE-014): a phase 3, double-blind, randomised, pla-cebo-controlled trial. Lancet Oncol. 2015;16:447-56.

195. Kruit WH, Suciu S, Dreno B, Mortier L, Robert C, Chiarion-Sileni V, et al. Selec-tion of immunostimulant AS15 for active immunization with MAGE-A3 protein: results of a randomized phase II study of the European Organisation for Research and Treatment of Cancer Melanoma Group in Metastatic Melanoma. J Clin Oncol. 2013;31:2413-20.

196. Kubota K, Hida T, Ishikura S, Mizusawa J, Nishio M, Kawahara M, et al. Etoposide and cisplatin versus irinotecan and cisplatin in patients with limited-stage small-cell lung cancer treated with etoposide and cisplatin plus concurrent accelerated hyperfractionated thoracic radiotherapy (JCOG0202): a randomised phase 3 study. Lancet Oncol. 2014;15:106-13.

197. Kunkler IH, Williams LJ, Jack WJ, Cameron DA, Dixon JM, investigators PI. Breast-conserving surgery with or without irradiation in women aged 65 years or older with early breast cancer (PRIME II): a randomised controlled trial. Lancet Oncol. 2015;16:266-73.

198. Kwon ED, Drake CG, Scher HI, Fizazi K, Bossi A, van den Eertwegh AJ, et al. Ipilimumab versus placebo after radiotherapy in patients with metastatic castra-tion-resistant prostate cancer that had progressed after docetaxel chemotherapy (CA184-043): a multicentre, randomised, double-blind, phase 3 trial. Lancet Oncol. 2014;15:700-12.

199. Lam WW, Chan M, Or A, Kwong A, Suen D, Fielding R. Reducing treatment decision conflict difficulties in breast cancer surgery: a randomized controlled trial. J Clin Oncol. 2013;31:2879-85.

200. Langer CJ, Novello S, Park K, Krzakowski M, Karp DD, Mok T, et al. Random-ized, phase III trial of first-line figitumumab in combination with paclitaxel and car-boplatin versus paclitaxel and carboplatin alone in patients with advanced non-small-cell lung cancer. J Clin Oncol. 2014;32:2059-66.

201. Langley RE, Cafferty FH, Alhasso AA, Rosen SD, Sundaram SK, Freeman SC, et al. Cardiovascular outcomes in patients with locally advanced and metastatic prostate cancer treated with luteinising-hormone-releasing-hormone agonists or transdermal oes-trogen: the randomised, phase 2 MRC PATCH trial (PR09). Lancet Oncol. 2013;14:306-16.

202. Larkin J, Ascierto PA, Dreno B, Atkinson V, Liszkay G, Maio M, et al. Combined vemurafenib and cobimetinib in BRAF-mutated melanoma. N Engl J Med. 2014;371:1867-76.

203. Larkin J, Chiarion-Sileni V, Gonzalez R, Grob JJ, Cowey CL, Lao CD, et al. Combined Nivolumab and Ipilimumab or Monotherapy in Untreated Melanoma. N Engl J Med. 2015;373:23-34.

204. Lawson DH, Lee S, Zhao F, Tarhini AA, Margolin KA, Ernstoff MS, et al. Ran-domized, Placebo-Controlled, Phase III Trial of Yeast-Derived Granulocyte-Macrophage Colony-Stimulating Factor (GM-CSF) Versus Peptide Vaccination Versus GM-CSF Plus Peptide Vaccination Versus Placebo in Patients With No Evidence of Disease After Com-plete Surgical Resection of Locally Advanced and/or Stage IV Melanoma: A Trial of the Eastern Cooperative Oncology Group-American College of Radiology Imaging Network Cancer Research Group (E4697). J Clin Oncol. 2015;33:4066-76.

205. Le Cesne A, Blay JY, Domont J, Tresch-Bruneel E, Chevreau C, Bertucci F, et al. Interruption versus continuation of trabectedin in patients with soft-tissue sarcoma (T-DIS): a randomised phase 2 trial. Lancet Oncol. 2015;16:312-9.

206. Le Deley MC, Paulussen M, Lewis I, Brennan B, Ranft A, Whelan J, et al. Cy-clophosphamide compared with ifosfamide in consolidation treatment of standard-risk Ewing sarcoma: results of the randomized noninferiority Euro-EWING99-R1 trial. J Clin Oncol. 2014;32:2440-8.

207. Le DT, Wang-Gillam A, Picozzi V, Greten TF, Crocenzi T, Springett G, et al. Safety and survival with GVAX pancreas prime and Listeria Monocytogenes-expressing mesothelin (CRS-207) boost vaccines for metastatic pancreatic cancer. J Clin Oncol. 2015;33:1325-33.

208. Leblond V, Johnson S, Chevret S, Copplestone A, Rule S, Tournilhac O, et al. Results of a randomized trial of chlorambucil versus fludarabine for patients with un-treated Waldenstrom macroglobulinemia, marginal zone lymphoma, or lympho-plasmacytic lymphoma. J Clin Oncol. 2013;31:301-7.

209. Lee JH, Joo YD, Kim H, Ryoo HM, Kim MK, Lee GW, et al. Randomized trial of myeloablative conditioning regimens: busulfan plus cyclophosphamide versus busulfan plus fludarabine. J Clin Oncol. 2013;31:701-9.

210. Lee SM, Lewanski CR, Counsell N, Ottensmeier C, Bates A, Patel N, et al. Ran-domized trial of erlotinib plus whole-brain radiotherapy for NSCLC patients with multi-ple brain metastases. J Natl Cancer Inst. 2014;106.

211. Leenstra JL, Miller RC, Qin R, Martenson JA, Dornfeld KJ, Bearden JD, et al. Doxepin rinse versus placebo in the treatment of acute oral mucositis pain in patients re-ceiving head and neck radiotherapy with or without chemotherapy: a phase III, random-ized, double-blind trial (NCCTG-N09C6 [Alliance]). J Clin Oncol. 2014;32:1571-7.

212. Lefebvre JL, Pointreau Y, Rolland F, Alfonsi M, Baudoux A, Sire C, et al. Induc-tion chemotherapy followed by either chemoradiotherapy or bioradiotherapy for larynx preservation: the TREMPLIN randomized phase II study. J Clin Oncol. 2013;31:853-9.

213. Leonard JP, Jung SH, Johnson J, Pitcher BN, Bartlett NL, Blum KA, et al. Ran-domized Trial of Lenalidomide Alone Versus Lenalidomide Plus Rituximab in Patients With Recurrent Follicular Lymphoma: CALGB 50401 (Alliance). J Clin Oncol. 2015;33:3635-40.

214. Lepore SJ, Buzaglo JS, Lieberman MA, Golant M, Greener JR, Davey A. Com-paring standard versus prosocial internet support groups for patients with breast cancer: a randomized controlled trial of the helper therapy principle. J Clin Oncol. 2014;32:4081-6.

215. Li J, Qin S, Xu J, Guo W, Xiong J, Bai Y, et al. Apatinib for chemothera-py-refractory advanced metastatic gastric cancer: results from a randomized, place-bo-controlled, parallel-arm, phase II trial. J Clin Oncol. 2013;31:3219-25.

216. Li J, Qin S, Xu R, Yau TC, Ma B, Pan H, et al. Regorafenib plus best supportive care versus placebo plus best supportive care in Asian patients with previously treated metastatic colorectal cancer (CONCUR): a randomised, double-blind, placebo-controlled, phase 3 trial. Lancet Oncol. 2015;16:619-29.

217. Liu JF, Barry WT, Birrer M, Lee JM, Buckanovich RJ, Fleming GF, et al. Combi-nation cediranib and olaparib versus olaparib alone for women with recurrent plati-num-sensitive ovarian cancer: a randomised phase 2 study. Lancet Oncol. 2014;15:1207-14.

218. Llovet JM, Decaens T, Raoul JL, Boucher E, Kudo M, Chang C, et al. Brivanib in patients with advanced hepatocellular carcinoma who were intolerant to sorafenib or for whom sorafenib failed: results from the randomized phase III BRISK-PS study. J Clin On-col. 2013;31:3509-16.

219. Lo-Coco F, Avvisati G, Vignetti M, Thiede C, Orlando SM, Iacobelli S, et al. Ret-inoic acid and arsenic trioxide for acute promyelocytic leukemia. N Engl J Med. 2013;369:111-21.

220. Long GV, Stroyakovskiy D, Gogas H, Levchenko E, de Braud F, Larkin J, et al. Combined BRAF and MEK inhibition versus BRAF inhibition alone in melanoma. N Engl J Med. 2014;371:1877-88.

221. Lonial S, Dimopoulos M, Palumbo A, White D, Grosicki S, Spicka I, et al. Elo-tuzumab Therapy for Relapsed or Refractory Multiple Myeloma. N Engl J Med. 2015;373:621-31.

222. Loprinzi CL, Qin R, Dakhil SR, Fehrenbacher L, Flynn KA, Atherton P, et al. Phase III randomized, placebo-controlled, double-blind study of intravenous calcium and magnesium to prevent oxaliplatin-induced sensory neurotoxicity (N08CB/Alliance). J Clin Oncol. 2014;32:997-1005.

223. Lordick F, Kang YK, Chung HC, Salman P, Oh SC, Bodoky G, et al. Capecita-bine and cisplatin with or without cetuximab for patients with previously untreated ad-vanced gastric cancer (EXPAND): a randomised, open-label phase 3 trial. Lancet Oncol. 2013;14:490-9.

224. Loriot Y, Miller K, Sternberg CN, Fizazi K, De Bono JS, Chowdhury S, et al. Ef-fect of enzalutamide on health-related quality of life, pain, and skeletal-related events in asymptomatic and minimally symptomatic, chemotherapy-naive patients with metastatic castration-resistant prostate cancer (PREVAIL): results from a randomised, phase 3 trial. Lancet Oncol. 2015;16:509-21.

225. Loupakis F, Cremolini C, Masi G, Lonardi S, Zagonel V, Salvatore L, et al. Ini-tial therapy with FOLFOXIRI and bevacizumab for metastatic colorectal cancer. N Engl J Med. 2014;371:1609-18.

226. Love RR, Laudico AV, Van Dinh N, Allred DC, Uy GB, Quang le H, et al. Tim-ing of adjuvant surgical oophorectomy in the menstrual cycle and disease-free and overall survival in premenopausal women with operable breast cancer. J Natl Cancer Inst. 2015;107:djv064.

227. Ludwig H, Viterbo L, Greil R, Masszi T, Spicka I, Shpilberg O, et al. Random-ized phase II study of bortezomib, thalidomide, and dexamethasone with or without cy-clophosphamide as induction therapy in previously untreated multiple myeloma. J Clin Oncol. 2013;31:247-55.

228. Machiels JP, Haddad RI, Fayette J, Licitra LF, Tahara M, Vermorken JB, et al. Afatinib versus methotrexate as second-line treatment in patients with recurrent or meta-static squamous-cell carcinoma of the head and neck progressing on or after plati-num-based therapy (LUX-Head & Neck 1): an open-label, randomised phase 3 trial. Lan-cet Oncol. 2015;16:583-94.

229. Mackey JR, Ramos-Vazquez M, Lipatov O, McCarthy N, Krasnozhon D, Semi-glazov V, et al. Primary results of ROSE/TRIO-12, a randomized placebo-controlled phase III trial evaluating the addition of ramucirumab to first-line docetaxel chemotherapy in metastatic breast cancer. J Clin Oncol. 2015;33:141-8.

230. Malka D, Cervera P, Foulon S, Trarbach T, de la Fouchardiere C, Boucher E, et al. Gemcitabine and oxaliplatin with or without cetuximab in advanced biliary-tract can-cer (BINGO): a randomised, open-label, non-comparative phase 2 trial. Lancet Oncol. 2014;15:819-28.

231. Mao JJ, Bowman MA, Xie SX, Bruner D, DeMichele A, Farrar JT. Electroacu-puncture Versus Gabapentin for Hot Flashes Among Breast Cancer Survivors: A Ran-domized Placebo-Controlled Trial. J Clin Oncol. 2015;33:3615-20.

232. Mariette C, Dahan L, Mornex F, Maillard E, Thomas PA, Meunier B, et al. Sur-gery alone versus chemoradiotherapy followed by surgery for stage I and II esophageal cancer: final analysis of randomized controlled phase III trial FFCD 9901. J Clin Oncol. 2014;32:2416-22.

233. Martin M, Loibl S, von Minckwitz G, Morales S, Martinez N, Guerrero A, et al. Phase III trial evaluating the addition of bevacizumab to endocrine therapy as first-line treatment for advanced breast cancer: the letrozole/fulvestrant and avastin (LEA) study. J Clin Oncol. 2015;33:1045-52.

234. Martin M, Ruiz A, Ruiz Borrego M, Barnadas A, Gonzalez S, Calvo L, et al. Fluorouracil, doxorubicin, and cyclophosphamide (FAC) versus FAC followed by weekly paclitaxel as adjuvant therapy for high-risk, node-negative breast cancer: results from the GEICAM/2003-02 study. J Clin Oncol. 2013;31:2593-9.

235. Martin M, Ruiz Simon A, Ruiz Borrego M, Ribelles N, Rodriguez-Lescure A, Munoz-Mateu M, et al. Epirubicin Plus Cyclophosphamide Followed by Docetaxel Versus Epirubicin Plus Docetaxel Followed by Capecitabine As Adjuvant Therapy for Node-Positive Early Breast Cancer: Results From the GEICAM/2003-10 Study. J Clin On-col. 2015;33:3788-95.

236. Martins RG, Parvathaneni U, Bauman JE, Sharma AK, Raez LE, Papagikos MA, et al. Cisplatin and radiotherapy with or without erlotinib in locally advanced squamous cell carcinoma of the head and neck: a randomized phase II trial. J Clin Oncol. 2013;31:1415-21.

237. Mason MD, Parulekar WR, Sydes MR, Brundage M, Kirkbride P, Gospo-darowicz M, et al. Final Report of the Intergroup Randomized Study of Combined Andro-gen-Deprivation Therapy Plus Radiotherapy Versus Androgen-Deprivation Therapy Alone in Locally Advanced Prostate Cancer. J Clin Oncol. 2015;33:2143-50.

238. Mateos MV, Hernandez MT, Giraldo P, de la Rubia J, de Arriba F, Lopez Corral L, et al. Lenalidomide plus dexamethasone for high-risk smoldering multiple myeloma. N Engl J Med. 2013;369:438-47.

239. Mayer RJ, Van Cutsem E, Falcone A, Yoshino T, Garcia-Carbonero R, Mizunu-ma N, et al. Randomized trial of TAS-102 for refractory metastatic colorectal cancer. N Engl J Med. 2015;372:1909-19.

240. McCormick B, Winter K, Hudis C, Kuerer HM, Rakovitch E, Smith BL, et al. RTOG 9804: a prospective randomized trial for good-risk ductal carcinoma in situ com-paring radiotherapy with observation. J Clin Oncol. 2015;33:709-15.

241. Mesia R, Henke M, Fortin A, Minn H, Yunes Ancona AC, Cmelak A, et al. Chemoradiotherapy with or without panitumumab in patients with unresected, locally advanced squamous-cell carcinoma of the head and neck (CONCERT-1): a randomised, controlled, open-label phase 2 trial. Lancet Oncol. 2015;16:208-20.

242. Michaelson MD, Oudard S, Ou YC, Sengelov L, Saad F, Houede N, et al. Ran-domized, placebo-controlled, phase III trial of sunitinib plus prednisone versus predni-sone alone in progressive, metastatic, castration-resistant prostate cancer. J Clin Oncol. 2014;32:76-82.

243. Middleton G, Silcocks P, Cox T, Valle J, Wadsley J, Propper D, et al. Gemcita-bine and capecitabine with or without telomerase peptide vaccine GV1001 in patients with locally advanced or metastatic pancreatic cancer (TeloVac): an open-label, random-ised, phase 3 trial. Lancet Oncol. 2014;15:829-40.

244. Migden MR, Guminski A, Gutzmer R, Dirix L, Lewis KD, Combemale P, et al. Treatment with two different doses of sonidegib in patients with locally advanced or met-astatic basal cell carcinoma (BOLT): a multicentre, randomised, double-blind phase 2 trial. Lancet Oncol. 2015;16:716-28.

245. Miguel JS, Weisel K, Moreau P, Lacy M, Song K, Delforge M, et al. Pomalido-mide plus low-dose dexamethasone versus high-dose dexamethasone alone for patients with relapsed and refractory multiple myeloma (MM-003): a randomised, open-label, phase 3 trial. Lancet Oncol. 2013;14:1055-66.

246. Milbury K, Spelman A, Wood C, Matin SF, Tannir N, Jonasch E, et al. Random-ized controlled trial of expressive writing for patients with renal cell carcinoma. J Clin Oncol. 2014;32:663-70.

247. Mitin T, Hunt D, Shipley WU, Kaufman DS, Uzzo R, Wu CL, et al. Tran-surethral surgery and twice-daily radiation plus paclitaxel-cisplatin or fluoroura-cil-cisplatin with selective bladder preservation and adjuvant chemotherapy for patients with muscle invasive bladder cancer (RTOG 0233): a randomised multicentre phase 2 tri-al. Lancet Oncol. 2013;14:863-72.

248. Mohr P, Hauschild A, Trefzer U, Enk A, Tilgen W, Loquai C, et al. Intermittent High-Dose Intravenous Interferon Alfa-2b for Adjuvant Treatment of Stage III Melanoma: Final Analysis of a Randomized Phase III Dermatologic Cooperative Oncology Group Tri-al. J Clin Oncol. 2015;33:4077-84.

249. Monk BJ, Poveda A, Vergote I, Raspagliesi F, Fujiwara K, Bae DS, et al. An-ti-angiopoietin therapy with trebananib for recurrent ovarian cancer (TRINOVA-1): a ran-domised, multicentre, double-blind, placebo-controlled phase 3 trial. Lancet Oncol. 2014;15:799-808.

250. Montgomery GH, David D, Kangas M, Green S, Sucala M, Bovbjerg DH, et al. Randomized controlled trial of a cognitive-behavioral therapy plus hypnosis intervention to control fatigue in patients undergoing radiotherapy for breast cancer. J Clin Oncol. 2014;32:557-63.

251. Moore HC, Unger JM, Phillips KA, Boyle F, Hitre E, Porter D, et al. Goserelin for ovarian protection during breast-cancer adjuvant chemotherapy. N Engl J Med. 2015;372:923-32.

252. Morschhauser FA, Cartron G, Thieblemont C, Solal-Celigny P, Haioun C, Bouabdallah R, et al. Obinutuzumab (GA101) monotherapy in relapsed/refractory diffuse large b-cell lymphoma or mantle-cell lymphoma: results from the phase II GAUGUIN study. J Clin Oncol. 2013;31:2912-9.

253. Morton DL, Thompson JF, Cochran AJ, Mozzillo N, Nieweg OE, Roses DF, et al. Final trial report of sentinel-node biopsy versus nodal observation in melanoma. N Engl J Med. 2014;370:599-609.

254. Motzer RJ, Barrios CH, Kim TM, Falcon S, Cosgriff T, Harker WG, et al. Phase II randomized trial comparing sequential first-line everolimus and second-line sunitinib versus first-line sunitinib and second-line everolimus in patients with metastatic renal cell carcinoma. J Clin Oncol. 2014;32:2765-72.

255. Motzer RJ, Escudier B, McDermott DF, George S, Hammers HJ, Srinivas S, et al. Nivolumab versus Everolimus in Advanced Renal-Cell Carcinoma. N Engl J Med. 2015;373:1803-13.

256. Motzer RJ, Hutson TE, Cella D, Reeves J, Hawkins R, Guo J, et al. Pazopanib versus sunitinib in metastatic renal-cell carcinoma. N Engl J Med. 2013;369:722-31.

257. Motzer RJ, Hutson TE, Glen H, Michaelson MD, Molina A, Eisen T, et al. Len-vatinib, everolimus, and the combination in patients with metastatic renal cell carcinoma: a randomised, phase 2, open-label, multicentre trial. Lancet Oncol. 2015;16:1473-82.

258. Motzer RJ, Nosov D, Eisen T, Bondarenko I, Lesovoy V, Lipatov O, et al. Tivozanib versus sorafenib as initial targeted therapy for patients with metastatic renal cell carcinoma: results from a phase III trial. J Clin Oncol. 2013;31:3791-9.

259. Motzer RJ, Porta C, Vogelzang NJ, Sternberg CN, Szczylik C, Zolnierek J, et al. Dovitinib versus sorafenib for third-line targeted treatment of patients with metastatic re-nal cell carcinoma: an open-label, randomised phase 3 trial. Lancet Oncol. 2014;15:286-96.

260. Motzer RJ, Rini BI, McDermott DF, Redman BG, Kuzel TM, Harrison MR, et al. Nivolumab for Metastatic Renal Cell Carcinoma: Results of a Randomized Phase II Trial. J Clin Oncol. 2015;33:1430-7.

261. Mukesh MB, Barnett GC, Wilkinson JS, Moody AM, Wilson C, Dorling L, et al. Randomized controlled trial of intensity-modulated radiotherapy for early breast cancer: 5-year results confirm superior overall cosmesis. J Clin Oncol. 2013;31:4488-95.

262. Mukherjee S, Hurt CN, Bridgewater J, Falk S, Cummins S, Wasan H, et al. Gem-citabine-based or capecitabine-based chemoradiotherapy for locally advanced pancreatic cancer (SCALOP): a multicentre, randomised, phase 2 trial. Lancet Oncol. 2013;14:317-26.

263. Mustian KM, Sprod LK, Janelsins M, Peppone LJ, Palesh OG, Chandwani K, et al. Multicenter, randomized controlled trial of yoga for sleep quality among cancer survi-vors. J Clin Oncol. 2013;31:3233-41.

264. Napolitano M, Saccullo G, Malato A, Sprini D, Ageno W, Imberti D, et al. Op-timal duration of low molecular weight heparin for the treatment of cancer-related deep vein thrombosis: the Cancer-DACUS Study. J Clin Oncol. 2014;32:3607-12.

265. Naumann RW, Coleman RL, Burger RA, Sausville EA, Kutarska E, Ghamande SA, et al. PRECEDENT: a randomized phase II trial comparing vintafolide (EC145) and pegylated liposomal doxorubicin (PLD) in combination versus PLD alone in patients with platinum-resistant ovarian cancer. J Clin Oncol. 2013;31:4400-6.

266. Nava S, Ferrer M, Esquinas A, Scala R, Groff P, Cosentini R, et al. Palliative use of non-invasive ventilation in end-of-life patients with solid tumours: a randomised feasi-bility trial. Lancet Oncol. 2013;14:219-27.

267. Niesvizky R, Flinn IW, Rifkin R, Gabrail N, Charu V, Clowney B, et al. Com-munity-Based Phase IIIB Trial of Three UPFRONT Bortezomib-Based Myeloma Regimens. J Clin Oncol. 2015;33:3921-9.

268. O'Day SJ, Eggermont AM, Chiarion-Sileni V, Kefford R, Grob JJ, Mortier L, et al. Final results of phase III SYMMETRY study: randomized, double-blind trial of elesclomol plus paclitaxel versus paclitaxel alone as treatment for chemotherapy-naive patients with advanced melanoma. J Clin Oncol. 2013;31:1211-8.

269. O'Shaughnessy J, Schwartzberg L, Danso MA, Miller KD, Rugo HS, Neubauer M, et al. Phase III study of iniparib plus gemcitabine and carboplatin versus gemcitabine and carboplatin in patients with metastatic triple-negative breast cancer. J Clin Oncol. 2014;32:3840-7.

270. Oettle H, Riess H, Stieler JM, Heil G, Schwaner I, Seraphin J, et al. Second-line oxaliplatin, folinic acid, and fluorouracil versus folinic acid and fluorouracil alone for gemcitabine-refractory pancreatic cancer: outcomes from the CONKO-003 trial. J Clin On-col. 2014;32:2423-9.

271. Ohtsu A, Ajani JA, Bai YX, Bang YJ, Chung HC, Pan HM, et al. Everolimus for previously treated advanced gastric cancer: results of the randomized, double-blind, phase III GRANITE-1 study. J Clin Oncol. 2013;31:3935-43.

272. Oza AM, Cibula D, Benzaquen AO, Poole C, Mathijssen RH, Sonke GS, et al. Olaparib combined with chemotherapy for recurrent platinum-sensitive ovarian cancer: a randomised phase 2 trial. Lancet Oncol. 2015;16:87-97.

273. Oza AM, Pignata S, Poveda A, McCormack M, Clamp A, Schwartz B, et al. Randomized Phase II Trial of Ridaforolimus in Advanced Endometrial Carcinoma. J Clin Oncol. 2015;33:3576-82.

274. Palmer JB, Lane D, Mayo D, Schluchter M, Leeming R. Effects of Music Therapy on Anesthesia Requirements and Anxiety in Women Undergoing Ambulatory Breast Surgery for Cancer Diagnosis and Treatment: A Randomized Controlled Trial. J Clin On-col. 2015;33:3162-8.

275. Palumbo A, Cavallo F, Gay F, Di Raimondo F, Ben Yehuda D, Petrucci MT, et al. Autologous transplantation and maintenance therapy in multiple myeloma. N Engl J Med. 2014;371:895-905.

276. Park SH, Sohn TS, Lee J, Lim DH, Hong ME, Kim KM, et al. Phase III Trial to Compare Adjuvant Chemotherapy With Capecitabine and Cisplatin Versus Concurrent Chemoradiotherapy in Gastric Cancer: Final Report of the Adjuvant Chemoradiotherapy in Stomach Tumors Trial, Including Survival and Subset Analyses. J Clin Oncol. 2015;33:3130-6.

277. Park YH, Jung KH, Im SA, Sohn JH, Ro J, Ahn JH, et al. Phase III, multicenter, randomized trial of maintenance chemotherapy versus observation in patients with met-astatic breast cancer after achieving disease control with six cycles of gemcitabine plus paclitaxel as first-line chemotherapy: KCSG-BR07-02. J Clin Oncol. 2013;31:1732-9.

278. Parker C, Nilsson S, Heinrich D, Helle SI, O'Sullivan JM, Fossa SD, et al. Alpha emitter radium-223 and survival in metastatic prostate cancer. N Engl J Med. 2013;369:213-23.

279. Patel JD, Socinski MA, Garon EB, Reynolds CH, Spigel DR, Olsen MR, et al. PointBreak: a randomized phase III study of pemetrexed plus carboplatin and bevaci-zumab followed by maintenance pemetrexed and bevacizumab versus paclitaxel plus carboplatin and bevacizumab followed by maintenance bevacizumab in patients with stage IIIB or IV nonsquamous non-small-cell lung cancer. J Clin Oncol. 2013;31:4349-57.

280. Paulsen O, Klepstad P, Rosland JH, Aass N, Albert E, Fayers P, et al. Efficacy of methylprednisolone on pain, fatigue, and appetite loss in patients with advanced cancer using opioids: a randomized, placebo-controlled, double-blind trial. J Clin Oncol. 2014;32:3221-8.

281. Payne MJ, Argyropoulou K, Lorigan P, McAleer JJ, Farrugia D, Davidson N, et al. Phase II pilot study of intravenous high-dose interferon with or without maintenance treatment in melanoma at high risk of recurrence. J Clin Oncol. 2014;32:185-90.

282. Paz-Ares L, Mezger J, Ciuleanu TE, Fischer JR, von Pawel J, Provencio M, et al. Necitumumab plus pemetrexed and cisplatin as first-line therapy in patients with stage IV non-squamous non-small-cell lung cancer (INSPIRE): an open-label, randomised, con-trolled phase 3 study. Lancet Oncol. 2015;16:328-37.

283. Pelzer U, Opitz B, Deutschinoff G, Stauch M, Reitzig PC, Hahnfeld S, et al. Effi-cacy of Prophylactic Low-Molecular Weight Heparin for Ambulatory Patients With Ad-vanced Pancreatic Cancer: Outcomes From the CONKO-004 Trial. J Clin Oncol. 2015;33:2028-34.

284. Peng ZW, Zhang YJ, Chen MS, Xu L, Liang HH, Lin XJ, et al. Radiofrequency ablation with or without transcatheter arterial chemoembolization in the treatment of hepatocellular carcinoma: a prospective randomized trial. J Clin Oncol. 2013;31:426-32.

285. Perez EA, Awada A, O'Shaughnessy J, Rugo HS, Twelves C, Im SA, et al. Etiri-notecan pegol (NKTR-102) versus treatment of physician's choice in women with ad-vanced breast cancer previously treated with an anthracycline, a taxane, and capecitabine (BEACON): a randomised, open-label, multicentre, phase 3 trial. Lancet Oncol. 2015;16:1556-68.

286. Petrylak DP, Vogelzang NJ, Budnik N, Wiechno PJ, Sternberg CN, Doner K, et al. Docetaxel and prednisone with or without lenalidomide in chemotherapy-naive pa-tients with metastatic castration-resistant prostate cancer (MAINSAIL): a randomised, double-blind, placebo-controlled phase 3 trial. Lancet Oncol. 2015;16:417-25.

287. Pettengell R, Schmitz N, Gisselbrecht C, Smith G, Patton WN, Metzner B, et al. Rituximab purging and/or maintenance in patients undergoing autologous transplanta-tion for relapsed follicular lymphoma: a prospective randomized trial from the lymphoma working party of the European group for blood and marrow transplantation. J Clin Oncol. 2013;31:1624-30.

288. Pignata S, Lorusso D, Scambia G, Sambataro D, Tamberi S, Cinieri S, et al. Pazopanib plus weekly paclitaxel versus weekly paclitaxel alone for platinum-resistant or platinum-refractory advanced ovarian cancer (MITO 11): a randomised, open-label, phase 2 trial. Lancet Oncol. 2015;16:561-8.

289. Pignata S, Scambia G, Katsaros D, Gallo C, Pujade-Lauraine E, De Placido S, et al. Carboplatin plus paclitaxel once a week versus every 3 weeks in patients with ad-vanced ovarian cancer (MITO-7): a randomised, multicentre, open-label, phase 3 trial. Lancet Oncol. 2014;15:396-405.

290. Pisansky TM, Hunt D, Gomella LG, Amin MB, Balogh AG, Chinn DM, et al. Duration of androgen suppression before radiotherapy for localized prostate cancer: radi-ation therapy oncology group randomized clinical trial 9910. J Clin Oncol. 2015;33:332-9.

291. Pivot X, Gligorov J, Muller V, Barrett-Lee P, Verma S, Knoop A, et al. Preference for subcutaneous or intravenous administration of trastuzumab in patients with HER2-positive early breast cancer (PrefHer): an open-label randomised study. Lancet On-col. 2013;14:962-70.

292. Pivot X, Manikhas A, Zurawski B, Chmielowska E, Karaszewska B, Allerton R, et al. CEREBEL (EGF111438): A Phase III, Randomized, Open-Label Study of Lapatinib Plus Capecitabine Versus Trastuzumab Plus Capecitabine in Patients With Human Epi-dermal Growth Factor Receptor 2-Positive Metastatic Breast Cancer. J Clin Oncol. 2015;33:1564-73.

293. Pivot X, Romieu G, Debled M, Pierga JY, Kerbrat P, Bachelot T, et al. 6 months versus 12 months of adjuvant trastuzumab for patients with HER2-positive early breast cancer (PHARE): a randomised phase 3 trial. Lancet Oncol. 2013;14:741-8.

294. Place AE, Stevenson KE, Vrooman LM, Harris MH, Hunt SK, O'Brien JE, et al. Intravenous pegylated asparaginase versus intramuscular native Escherichia coli L-asparaginase in newly diagnosed childhood acute lymphoblastic leukaemia (DFCI 05-001): a randomised, open-label phase 3 trial. Lancet Oncol. 2015;16:1677-90.

295. Pollack A, Walker G, Horwitz EM, Price R, Feigenberg S, Konski AA, et al. Randomized trial of hypofractionated external-beam radiotherapy for prostate cancer. J Clin Oncol. 2013;31:3860-8.

296. Poortmans PM, Collette S, Kirkove C, Van Limbergen E, Budach V, Struikmans H, et al. Internal Mammary and Medial Supraclavicular Irradiation in Breast Cancer. N Engl J Med. 2015;373:317-27.

297. Poplin E, Wasan H, Rolfe L, Raponi M, Ikdahl T, Bondarenko I, et al. Random-ized, multicenter, phase II study of CO-101 versus gemcitabine in patients with metastatic pancreatic ductal adenocarcinoma: including a prospective evaluation of the role of hENT1 in gemcitabine or CO-101 sensitivity. J Clin Oncol. 2013;31:4453-61.

298. Postow MA, Chesney J, Pavlick AC, Robert C, Grossmann K, McDermott D, et al. Nivolumab and ipilimumab versus ipilimumab in untreated melanoma. N Engl J Med. 2015;372:2006-17.

299. Prebet T, Sun Z, Figueroa ME, Ketterling R, Melnick A, Greenberg PL, et al. Pro-longed administration of azacitidine with or without entinostat for myelodysplastic syn-drome and acute myeloid leukemia with myelodysplasia-related changes: results of the US Leukemia Intergroup trial E1905. J Clin Oncol. 2014;32:1242-8.

300. Press OW, Unger JM, Rimsza LM, Friedberg JW, LeBlanc M, Czuczman MS, et al. Phase III randomized intergroup trial of CHOP plus rituximab compared with CHOP chemotherapy plus (131)iodine-tositumomab for previously untreated follicular non-Hodgkin lymphoma: SWOG S0016. J Clin Oncol. 2013;31:314-20.

301. Price TJ, Peeters M, Kim TW, Li J, Cascinu S, Ruff P, et al. Panitumumab versus cetuximab in patients with chemotherapy-refractory wild-type KRAS exon 2 metastatic colorectal cancer (ASPECCT): a randomised, multicentre, open-label, non-inferiority phase 3 study. Lancet Oncol. 2014;15:569-79.

302. Primrose J, Falk S, Finch-Jones M, Valle J, O'Reilly D, Siriwardena A, et al. Sys-temic chemotherapy with or without cetuximab in patients with resectable colorectal liver metastasis: the New EPOC randomised controlled trial. Lancet Oncol. 2014;15:601-11.

303. Pujade-Lauraine E, Hilpert F, Weber B, Reuss A, Poveda A, Kristensen G, et al. Bevacizumab combined with chemotherapy for platinum-resistant recurrent ovarian cancer: The AURELIA open-label randomized phase III trial. J Clin Oncol. 2014;32:1302-8.

304. Qin S, Bai Y, Lim HY, Thongprasert S, Chao Y, Fan J, et al. Randomized, multi-center, open-label study of oxaliplatin plus fluorouracil/leucovorin versus doxorubicin as palliative chemotherapy in patients with advanced hepatocellular carcinoma from Asia. J Clin Oncol. 2013;31:3501-8.

305. Quinn DI, Tangen CM, Hussain M, Lara PN, Jr., Goldkorn A, Moinpour CM, et al. Docetaxel and atrasentan versus docetaxel and placebo for men with advanced castra-tion-resistant prostate cancer (SWOG S0421): a randomised phase 3 trial. Lancet Oncol. 2013;14:893-900.

306. Quoix E, Lena H, Losonczy G, Forget F, Chouaid C, Papai Z, et al. TG4010 im-munotherapy and first-line chemotherapy for advanced non-small-cell lung cancer (TIME): results from the phase 2b part of a randomised, double-blind, placebo-controlled, phase 2b/3 trial. Lancet Oncol. 2016;17:212-23.

307. Radford J, Illidge T, Counsell N, Hancock B, Pettengell R, Johnson P, et al. Re-sults of a trial of PET-directed therapy for early-stage Hodgkin's lymphoma. N Engl J Med. 2015;372:1598-607.

308. Ramalingam SS, Janne PA, Mok T, O'Byrne K, Boyer MJ, Von Pawel J, et al. Dacomitinib versus erlotinib in patients with advanced-stage, previously treated non-small-cell lung cancer (ARCHER 1009): a randomised, double-blind, phase 3 trial. Lancet Oncol. 2014;15:1369-78.

309. Ramalingam SS, Shtivelband M, Soo RA, Barrios CH, Makhson A, Segalla JG, et al. Randomized phase II study of carboplatin and paclitaxel with either linifanib or pla-cebo for advanced nonsquamous non-small-cell lung cancer. J Clin Oncol. 2015;33:433-41.

310. Rambaldi A, Grassi A, Masciulli A, Boschini C, Mico MC, Busca A, et al. Busulfan plus cyclophosphamide versus busulfan plus fludarabine as a preparative reg-imen for allogeneic haemopoietic stem-cell transplantation in patients with acute myeloid leukaemia: an open-label, multicentre, randomised, phase 3 trial. Lancet Oncol. 2015;16:1525-36.

311. Rapp SR, Case LD, Peiffer A, Naughton MM, Chan MD, Stieber VW, et al. Donepezil for Irradiated Brain Tumor Survivors: A Phase III Randomized Place-bo-Controlled Clinical Trial. J Clin Oncol. 2015;33:1653-9.

312. Ravandi F, Ritchie EK, Sayar H, Lancet JE, Craig MD, Vey N, et al. Vosaroxin plus cytarabine versus placebo plus cytarabine in patients with first relapsed or refractory acute myeloid leukaemia (VALOR): a randomised, controlled, double-blind, multination-al, phase 3 study. Lancet Oncol. 2015;16:1025-36.

313. Ray-Coquard IL, Domont J, Tresch-Bruneel E, Bompas E, Cassier PA, Mir O, et al. Paclitaxel Given Once Per Week With or Without Bevacizumab in Patients With Ad-vanced Angiosarcoma: A Randomized Phase II Trial. J Clin Oncol. 2015;33:2797-802.

314. Ready NE, Pang HH, Gu L, Otterson GA, Thomas SP, Miller AA, et al. Chemo-therapy With or Without Maintenance Sunitinib for Untreated Extensive-Stage Small-Cell Lung Cancer: A Randomized, Double-Blind, Placebo-Controlled Phase II Study-CALGB 30504 (Alliance). J Clin Oncol. 2015;33:1660-5.

315. Reck M, Kaiser R, Mellemgaard A, Douillard JY, Orlov S, Krzakowski M, et al. Docetaxel plus nintedanib versus docetaxel plus placebo in patients with previously treated non-small-cell lung cancer (LUME-Lung 1): a phase 3, double-blind, randomised controlled trial. Lancet Oncol. 2014;15:143-55.

316. Ren Z, Zhu K, Kang H, Lu M, Qu Z, Lu L, et al. Randomized controlled trial of the prophylactic effect of urea-based cream on sorafenib-associated hand-foot skin reac-tions in patients with advanced hepatocellular carcinoma. J Clin Oncol. 2015;33:894-900.

317. Ribas A, Kefford R, Marshall MA, Punt CJ, Haanen JB, Marmol M, et al. Phase III randomized clinical trial comparing tremelimumab with standard-of-care chemother-apy in patients with advanced melanoma. J Clin Oncol. 2013;31:616-22.

318. Ribas A, Puzanov I, Dummer R, Schadendorf D, Hamid O, Robert C, et al. Pembrolizumab versus investigator-choice chemotherapy for ipilimumab-refractory mel-anoma (KEYNOTE-002): a randomised, controlled, phase 2 trial. Lancet Oncol. 2015;16:908-18.

319. Ribera JM, Oriol A, Morgades M, Montesinos P, Sarra J, Gonzalez-Campos J, et al. Treatment of high-risk Philadelphia chromosome-negative acute lymphoblastic leu-kemia in adolescents and adults according to early cytologic response and minimal re-sidual disease after consolidation assessed by flow cytometry: final results of the PETHEMA ALL-AR-03 trial. J Clin Oncol. 2014;32:1595-604.

320. Rini BI, Bellmunt J, Clancy J, Wang K, Niethammer AG, Hariharan S, et al. Randomized phase III trial of temsirolimus and bevacizumab versus interferon alfa and bevacizumab in metastatic renal cell carcinoma: INTORACT trial. J Clin Oncol. 2014;32:752-9.

321. Rini BI, Melichar B, Ueda T, Grunwald V, Fishman MN, Arranz JA, et al. Ax-itinib with or without dose titration for first-line metastatic renal-cell carcinoma: a ran-domised double-blind phase 2 trial. Lancet Oncol. 2013;14:1233-42.

322. Roa W, Kepka L, Kumar N, Sinaika V, Matiello J, Lomidze D, et al. International Atomic Energy Agency Randomized Phase III Study of Radiation Therapy in Elderly and/or Frail Patients With Newly Diagnosed Glioblastoma Multiforme. J Clin Oncol. 2015;33:4145-50.

323. Robak T, Huang H, Jin J, Zhu J, Liu T, Samoilova O, et al. Bortezomib-based therapy for newly diagnosed mantle-cell lymphoma. N Engl J Med. 2015;372:944-53.

324. Robert C, Dummer R, Gutzmer R, Lorigan P, Kim KB, Nyakas M, et al. Selu-metinib plus dacarbazine versus placebo plus dacarbazine as first-line treatment for BRAF-mutant metastatic melanoma: a phase 2 double-blind randomised study. Lancet Oncol. 2013;14:733-40.

325. Robert C, Karaszewska B, Schachter J, Rutkowski P, Mackiewicz A, Stroiakov-ski D, et al. Improved overall survival in melanoma with combined dabrafenib and tra-metinib. N Engl J Med. 2015;372:30-9.

326. Robert C, Long GV, Brady B, Dutriaux C, Maio M, Mortier L, et al. Nivolumab in previously untreated melanoma without BRAF mutation. N Engl J Med. 2015;372:320-30.

327. Robert C, Schachter J, Long GV, Arance A, Grob JJ, Mortier L, et al. Pembroli-zumab versus Ipilimumab in Advanced Melanoma. N Engl J Med. 2015;372:2521-32.

328. Robertson JF, Ferrero JM, Bourgeois H, Kennecke H, de Boer RH, Jacot W, et al. Ganitumab with either exemestane or fulvestrant for postmenopausal women with ad-vanced, hormone-receptor-positive breast cancer: a randomised, controlled, double-blind, phase 2 trial. Lancet Oncol. 2013;14:228-35.

329. Robidoux A, Tang G, Rastogi P, Geyer CE, Jr., Azar CA, Atkins JN, et al. Lapa-tinib as a component of neoadjuvant therapy for HER2-positive operable breast cancer (NSABP protocol B-41): an open-label, randomised phase 3 trial. Lancet Oncol. 2013;14:1183-92.

330. Roboz GJ, Rosenblat T, Arellano M, Gobbi M, Altman JK, Montesinos P, et al. International randomized phase III study of elacytarabine versus investigator choice in patients with relapsed/refractory acute myeloid leukemia. J Clin Oncol. 2014;32:1919-26.

331. Rodel C, Graeven U, Fietkau R, Hohenberger W, Hothorn T, Arnold D, et al. Oxaliplatin added to fluorouracil-based preoperative chemoradiotherapy and postopera-tive chemotherapy of locally advanced rectal cancer (the German CAO/ARO/AIO-04 study): final results of the multicentre, open-label, randomised, phase 3 trial. Lancet On-col. 2015;16:979-89.

332. Roila F, Ruggeri B, Ballatori E, Del Favero A, Tonato M. Aprepitant versus dexamethasone for preventing chemotherapy-induced delayed emesis in patients with breast cancer: a randomized double-blind study. J Clin Oncol. 2014;32:101-6.

333. Rollig C, Serve H, Huttmann A, Noppeney R, Muller-Tidow C, Krug U, et al. Addition of sorafenib versus placebo to standard therapy in patients aged 60 years or younger with newly diagnosed acute myeloid leukaemia (SORAML): a multicentre, phase 2, randomised controlled trial. Lancet Oncol. 2015;16:1691-9.

334. Roscoe JA, Garland SN, Heckler CE, Perlis ML, Peoples AR, Shayne M, et al. Randomized placebo-controlled trial of cognitive behavioral therapy and armodafinil for insomnia after cancer treatment. J Clin Oncol. 2015;33:165-71.

335. Rugo HS, Barry WT, Moreno-Aspitia A, Lyss AP, Cirrincione C, Leung E, et al. Randomized Phase III Trial of Paclitaxel Once Per Week Compared With Nanoparticle Albumin-Bound Nab-Paclitaxel Once Per Week or Ixabepilone With Bevacizumab As First-Line Chemotherapy for Locally Recurrent or Metastatic Breast Cancer: CALGB 40502/NCCTG N063H (Alliance). J Clin Oncol. 2015;33:2361-9.

336. Ryan CJ, Smith MR, de Bono JS, Molina A, Logothetis CJ, de Souza P, et al. Abi-raterone in metastatic prostate cancer without previous chemotherapy. N Engl J Med. 2013;368:138-48.

337. Ryan CJ, Smith MR, Fizazi K, Saad F, Mulders PF, Sternberg CN, et al. Abi-raterone acetate plus prednisone versus placebo plus prednisone in chemotherapy-naive men with metastatic castration-resistant prostate cancer (COU-AA-302): final overall sur-vival analysis of a randomised, double-blind, placebo-controlled phase 3 study. Lancet Oncol. 2015;16:152-60.

338. Saad F, Fizazi K, Jinga V, Efstathiou E, Fong PC, Hart LL, et al. Orteronel plus prednisone in patients with chemotherapy-naive metastatic castration-resistant prostate cancer (ELM-PC 4): a double-blind, multicentre, phase 3, randomised, placebo-controlled trial. Lancet Oncol. 2015;16:338-48.

339. Sabbatini P, Harter P, Scambia G, Sehouli J, Meier W, Wimberger P, et al. Aba-govomab as maintenance therapy in patients with epithelial ovarian cancer: a phase III trial of the AGO OVAR, COGI, GINECO, and GEICO--the MIMOSA study. J Clin Oncol. 2013;31:1554-61.

340. Salles GA, Morschhauser F, Solal-Celigny P, Thieblemont C, Lamy T, Tilly H, et al. Obinutuzumab (GA101) in patients with relapsed/refractory indolent non-Hodgkin lymphoma: results from the phase II GAUGUIN study. J Clin Oncol. 2013;31:2920-6.

341. San-Miguel JF, Hungria VT, Yoon SS, Beksac M, Dimopoulos MA, Elghandour A, et al. Panobinostat plus bortezomib and dexamethasone versus placebo plus borte-zomib and dexamethasone in patients with relapsed or relapsed and refractory multiple myeloma: a multicentre, randomised, double-blind phase 3 trial. Lancet Oncol. 2014;15:1195-206.

342. Santoro A, Rimassa L, Borbath I, Daniele B, Salvagni S, Van Laethem JL, et al. Tivantinib for second-line treatment of advanced hepatocellular carcinoma: a random-ised, placebo-controlled phase 2 study. Lancet Oncol. 2013;14:55-63.

343. Sartor O, Coleman R, Nilsson S, Heinrich D, Helle SI, O'Sullivan JM, et al. Effect of radium-223 dichloride on symptomatic skeletal events in patients with castra-tion-resistant prostate cancer and bone metastases: results from a phase 3, double-blind, randomised trial. Lancet Oncol. 2014;15:738-46.

344. Satoh T, Xu RH, Chung HC, Sun GP, Doi T, Xu JM, et al. Lapatinib plus paclitaxel versus paclitaxel alone in the second-line treatment of HER2-amplified ad-vanced gastric cancer in Asian populations: TyTAN--a randomized, phase III study. J Clin Oncol. 2014;32:2039-49.

345. Satouchi M, Kotani Y, Shibata T, Ando M, Nakagawa K, Yamamoto N, et al. Phase III study comparing amrubicin plus cisplatin with irinotecan plus cisplatin in the treatment of extensive-disease small-cell lung cancer: JCOG 0509. J Clin Oncol. 2014;32:1262-8.

346. Scagliotti G, von Pawel J, Novello S, Ramlau R, Favaretto A, Barlesi F, et al. Phase III Multinational, Randomized, Double-Blind, Placebo-Controlled Study of Tivantinib (ARQ 197) Plus Erlotinib Versus Erlotinib Alone in Previously Treated Patients With Locally Advanced or Metastatic Nonsquamous Non-Small-Cell Lung Cancer. J Clin Oncol. 2015;33:2667-74.

347. Schaich M, Parmentier S, Kramer M, Illmer T, Stolzel F, Rollig C, et al. High-dose cytarabine consolidation with or without additional amsacrine and mitoxan-trone in acute myeloid leukemia: results of the prospective randomized AML2003 trial. J Clin Oncol. 2013;31:2094-102.

348. Schlumberger M, Tahara M, Wirth LJ, Robinson B, Brose MS, Elisei R, et al. Lenvatinib versus placebo in radioiodine-refractory thyroid cancer. N Engl J Med. 2015;372:621-30.

349. Schmitt T, Goldschmidt H, Neben K, Freiberger A, Husing J, Gronkowski M, et al. Aprepitant, granisetron, and dexamethasone for prevention of chemotherapy-induced nausea and vomiting after high-dose melphalan in autologous transplantation for multi-ple myeloma: results of a randomized, placebo-controlled phase III trial. J Clin Oncol. 2014;32:3413-20.

350. Schmoll HJ, Tabernero J, Maroun J, de Braud F, Price T, Van Cutsem E, et al. Capecitabine Plus Oxaliplatin Compared With Fluorouracil/Folinic Acid As Adjuvant Therapy for Stage III Colon Cancer: Final Results of the NO16968 Randomized Controlled Phase III Trial. J Clin Oncol. 2015;33:3733-40.

351. Schwartzberg LS, Modiano MR, Rapoport BL, Chasen MR, Gridelli C, Urban L, et al. Safety and efficacy of rolapitant for prevention of chemotherapy-induced nausea and vomiting after administration of moderately emetogenic chemotherapy or anthracycline and cyclophosphamide regimens in patients with cancer: a randomised, active-controlled, double-blind, phase 3 trial. Lancet Oncol. 2015;16:1071-8.

352. Schwartzberg LS, Rivera F, Karthaus M, Fasola G, Canon JL, Hecht JR, et al. PEAK: a randomized, multicenter phase II study of panitumumab plus modified fluor-ouracil, leucovorin, and oxaliplatin (mFOLFOX6) or bevacizumab plus mFOLFOX6 in pa-tients with previously untreated, unresectable, wild-type KRAS exon 2 metastatic colorec-tal cancer. J Clin Oncol. 2014;32:2240-7.

353. Sclafani F, Kim TY, Cunningham D, Kim TW, Tabernero J, Schmoll HJ, et al. A Randomized Phase II/III Study of Dalotuzumab in Combination With Cetuximab and Iri-notecan in Chemorefractory, KRAS Wild-Type, Metastatic Colorectal Cancer. J Natl Cancer Inst. 2015;107:djv258.

354. Sehn LH, Goy A, Offner FC, Martinelli G, Caballero MD, Gadeberg O, et al. Randomized Phase II Trial Comparing Obinutuzumab (GA101) With Rituximab in Pa-tients With Relapsed CD20+ Indolent B-Cell Non-Hodgkin Lymphoma: Final Analysis of the GAUSS Study. J Clin Oncol. 2015;33:3467-74.

355. Sequist LV, Yang JC, Yamamoto N, O'Byrne K, Hirsh V, Mok T, et al. Phase III study of afatinib or cisplatin plus pemetrexed in patients with metastatic lung adenocar-cinoma with EGFR mutations. J Clin Oncol. 2013;31:3327-34.

356. Serve H, Krug U, Wagner R, Sauerland MC, Heinecke A, Brunnberg U, et al. Sorafenib in combination with intensive chemotherapy in elderly patients with acute my-eloid leukemia: results from a randomized, placebo-controlled trial. J Clin Oncol. 2013;31:3110-8.

357. Seto T, Kato T, Nishio M, Goto K, Atagi S, Hosomi Y, et al. Erlotinib alone or with bevacizumab as first-line therapy in patients with advanced non-squamous non-small-cell lung cancer harbouring EGFR mutations (JO25567): an open-label, ran-domised, multicentre, phase 2 study. Lancet Oncol. 2014;15:1236-44.

358. Seymour MT, Brown SR, Middleton G, Maughan T, Richman S, Gwyther S, et al. Panitumumab and irinotecan versus irinotecan alone for patients with KRAS wild-type, fluorouracil-resistant advanced colorectal cancer (PICCOLO): a prospectively stratified randomised trial. Lancet Oncol. 2013;14:749-59.

359. Shah MA, Janjigian YY, Stoller R, Shibata S, Kemeny M, Krishnamurthi S, et al. Randomized Multicenter Phase II Study of Modified Docetaxel, Cisplatin, and Fluoroura-cil (DCF) Versus DCF Plus Growth Factor Support in Patients With Metastatic Gastric Adenocarcinoma: A Study of the US Gastric Cancer Consortium. J Clin Oncol. 2015;33:3874-9.

360. Shaw AT, Kim DW, Nakagawa K, Seto T, Crino L, Ahn MJ, et al. Crizotinib versus chemotherapy in advanced ALK-positive lung cancer. N Engl J Med. 2013;368:2385-94.

361. Shi M, Lu LG, Fang WQ, Guo RP, Chen MS, Li Y, et al. Roles played by chemolipiodolization and embolization in chemoembolization for hepatocellular carci-noma: single-blind, randomized trial. J Natl Cancer Inst. 2013;105:59-68.

362. Shi Y, Zhang L, Liu X, Zhou C, Zhang L, Zhang S, et al. Icotinib versus gefitinib in previously treated advanced non-small-cell lung cancer (ICOGEN): a randomised, double-blind phase 3 non-inferiority trial. Lancet Oncol. 2013;14:953-61.

363. Shike M, Doane AS, Russo L, Cabal R, Reis-Filho JS, Gerald W, et al. The effects of soy supplementation on gene expression in breast cancer: a randomized place-bo-controlled study. J Natl Cancer Inst. 2014;106.

364. Shinagawa K, Yanada M, Sakura T, Ueda Y, Sawa M, Miyatake J, et al. Ta-mibarotene as maintenance therapy for acute promyelocytic leukemia: results from a randomized controlled trial. J Clin Oncol. 2014;32:3729-35.

365. Shore ND, Chowdhury S, Villers A, Klotz L, Siemens DR, Phung D, et al. Effi-cacy and safety of enzalutamide versus bicalutamide for patients with metastatic prostate cancer (TERRAIN): a randomised, double-blind, phase 2 study. Lancet Oncol. 2016;17:153-63.

366. Shukuya T, Yamanaka T, Seto T, Daga H, Goto K, Saka H, et al. Nedaplatin plus docetaxel versus cisplatin plus docetaxel for advanced or relapsed squamous cell carcinoma of the lung (WJOG5208L): a randomised, open-label, phase 3 trial. Lancet On-col. 2015;16:1630-8.

367. Shulman LN, Berry DA, Cirrincione CT, Becker HP, Perez EA, O'Regan R, et al. Comparison of doxorubicin and cyclophosphamide versus single-agent paclitaxel as ad-juvant therapy for breast cancer in women with 0 to 3 positive axillary nodes: CALGB 40101 (Alliance). J Clin Oncol. 2014;32:2311-7.

368. Sikov WM, Berry DA, Perou CM, Singh B, Cirrincione CT, Tolaney SM, et al. Impact of the addition of carboplatin and/or bevacizumab to neoadjuvant once-per-week paclitaxel followed by dose-dense doxorubicin and cyclophosphamide on pathologic complete response rates in stage II to III triple-negative breast cancer: CALGB 40603 (Alli-ance). J Clin Oncol. 2015;33:13-21.

369. Siu LL, Shapiro JD, Jonker DJ, Karapetis CS, Zalcberg JR, Simes J, et al. Phase III randomized, placebo-controlled study of cetuximab plus brivanib alaninate versus ce-tuximab plus placebo in patients with metastatic, chemotherapy-refractory, wild-type K-RAS colorectal carcinoma: the NCIC Clinical Trials Group and AGITG CO.20 Trial. J Clin Oncol. 2013;31:2477-84.

370. Smerage JB, Barlow WE, Hortobagyi GN, Winer EP, Leyland-Jones B, Srkalovic G, et al. Circulating tumor cells and response to chemotherapy in metastatic breast cancer: SWOG S0500. J Clin Oncol. 2014;32:3483-9.

371. Smith DC, Smith MR, Sweeney C, Elfiky AA, Logothetis C, Corn PG, et al. Cabozantinib in patients with advanced prostate cancer: results of a phase II randomized discontinuation trial. J Clin Oncol. 2013;31:412-9.

372. Smith MR, Halabi S, Ryan CJ, Hussain A, Vogelzang N, Stadler W, et al. Ran-domized controlled trial of early zoledronic acid in men with castration-sensitive prostate cancer and bone metastases: results of CALGB 90202 (alliance). J Clin Oncol. 2014;32:1143-50.

373. Solomon BJ, Mok T, Kim DW, Wu YL, Nakagawa K, Mekhail T, et al. First-line crizotinib versus chemotherapy in ALK-positive lung cancer. N Engl J Med. 2014;371:2167-77.

374. Soria JC, Felip E, Cobo M, Lu S, Syrigos K, Lee KH, et al. Afatinib versus erlo-tinib as second-line treatment of patients with advanced squamous cell carcinoma of the lung (LUX-Lung 8): an open-label randomised controlled phase 3 trial. Lancet Oncol. 2015;16:897-907.

375. Soria JC, Wu YL, Nakagawa K, Kim SW, Yang JJ, Ahn MJ, et al. Gefitinib plus chemotherapy versus placebo plus chemotherapy in EGFR-mutation-positive non-small-cell lung cancer after progression on first-line gefitinib (IMPRESS): a phase 3 randomised trial. Lancet Oncol. 2015;16:990-8.

376. Spathis A, Fife K, Blackhall F, Dutton S, Bahadori R, Wharton R, et al. Modafinil for the treatment of fatigue in lung cancer: results of a placebo-controlled, double-blind, randomized trial. J Clin Oncol. 2014;32:1882-8.

377. Spigel DR, Ervin TJ, Ramlau RA, Daniel DB, Goldschmidt JH, Jr., Blu-menschein GR, Jr., et al. Randomized phase II trial of Onartuzumab in combination with erlotinib in patients with advanced non-small-cell lung cancer. J Clin Oncol. 2013;31:4105-14.

378. Stahel RA, Riesterer O, Xyrafas A, Opitz I, Beyeler M, Ochsenbein A, et al. Ne-oadjuvant chemotherapy and extrapleural pneumonectomy of malignant pleural meso-thelioma with or without hemithoracic radiotherapy (SAKK 17/04): a randomised, inter-national, multicentre phase 2 trial. Lancet Oncol. 2015;16:1651-8.

379. Stanworth SJ, Estcourt LJ, Powter G, Kahan BC, Dyer C, Choo L, et al. A no-prophylaxis platelet-transfusion strategy for hematologic cancers. N Engl J Med. 2013;368:1771-80.

380. Stary J, Zimmermann M, Campbell M, Castillo L, Dibar E, Donska S, et al. In-tensive chemotherapy for childhood acute lymphoblastic leukemia: results of the ran-domized intercontinental trial ALL IC-BFM 2002. J Clin Oncol. 2014;32:174-84.

381. Sternberg CN, Skoneczna I, Kerst JM, Albers P, Fossa SD, Agerbaek M, et al. Immediate versus deferred chemotherapy after radical cystectomy in patients with pT3-pT4 or N+ M0 urothelial carcinoma of the bladder (EORTC 30994): an intergroup, open-label, randomised phase 3 trial. Lancet Oncol. 2015;16:76-86.

382. Stewart AK, Rajkumar SV, Dimopoulos MA, Masszi T, Spicka I, Oriol A, et al. Carfilzomib, lenalidomide, and dexamethasone for relapsed multiple myeloma. N Engl J Med. 2015;372:142-52.

383. Stiff PJ, Unger JM, Cook JR, Constine LS, Couban S, Stewart DA, et al. Autolo-gous transplantation as consolidation for aggressive non-Hodgkin's lymphoma. N Engl J Med. 2013;369:1681-90.

384. Stone RM, Mazzola E, Neuberg D, Allen SL, Pigneux A, Stuart RK, et al. Phase III open-label randomized study of cytarabine in combination with amonafide L-malate or daunorubicin as induction therapy for patients with secondary acute myeloid leukemia. J Clin Oncol. 2015;33:1252-7.

385. Stupp R, Hegi ME, Gorlia T, Erridge SC, Perry J, Hong YK, et al. Cilengitide combined with standard treatment for patients with newly diagnosed glioblastoma with methylated MGMT promoter (CENTRIC EORTC 26071-22072 study): a multicentre, ran-domised, open-label, phase 3 trial. Lancet Oncol. 2014;15:1100-8.

386. Sun JM, Ahn JS, Jung SH, Sun J, Ha SY, Han J, et al. Pemetrexed Plus Cisplatin Versus Gemcitabine Plus Cisplatin According to Thymidylate Synthase Expression in Nonsquamous Non-Small-Cell Lung Cancer: A Biomarker-Stratified Randomized Phase II Trial. J Clin Oncol. 2015;33:2450-6.

387. Swain SM, Baselga J, Kim SB, Ro J, Semiglazov V, Campone M, et al. Per-tuzumab, trastuzumab, and docetaxel in HER2-positive metastatic breast cancer. N Engl J Med. 2015;372:724-34.

388. Swain SM, Tang G, Geyer CE, Jr., Rastogi P, Atkins JN, Donnellan PP, et al. De-finitive results of a phase III adjuvant trial comparing three chemotherapy regimens in women with operable, node-positive breast cancer: the NSABP B-38 trial. J Clin Oncol. 2013;31:3197-204.

389. Sweeney CJ, Chen YH, Carducci M, Liu G, Jarrard DF, Eisenberger M, et al. Chemohormonal Therapy in Metastatic Hormone-Sensitive Prostate Cancer. N Engl J Med. 2015;373:737-46.

390. Symonds RP, Gourley C, Davidson S, Carty K, McCartney E, Rai D, et al. Cediranib combined with carboplatin and paclitaxel in patients with metastatic or recur-rent cervical cancer (CIRCCa): a randomised, double-blind, placebo-controlled phase 2 tri-al. Lancet Oncol. 2015;16:1515-24.

391. Taal W, Oosterkamp HM, Walenkamp AM, Dubbink HJ, Beerepoot LV, Hanse MC, et al. Single-agent bevacizumab or lomustine versus a combination of bevacizumab plus lomustine in patients with recurrent glioblastoma (BELOB trial): a randomised con-trolled phase 2 trial. Lancet Oncol. 2014;15:943-53.

392. Tabernero J, Yoshino T, Cohn AL, Obermannova R, Bodoky G, Gar-cia-Carbonero R, et al. Ramucirumab versus placebo in combination with second-line FOLFIRI in patients with metastatic colorectal carcinoma that progressed during or after first-line therapy with bevacizumab, oxaliplatin, and a fluoropyrimidine (RAISE): a ran-domised, double-blind, multicentre, phase 3 study. Lancet Oncol. 2015;16:499-508.

393. Taieb J, Tabernero J, Mini E, Subtil F, Folprecht G, Van Laethem JL, et al. Oxali-platin, fluorouracil, and leucovorin with or without cetuximab in patients with resected stage III colon cancer (PETACC-8): an open-label, randomised phase 3 trial. Lancet Oncol. 2014;15:862-73.

394. Tannock IF, Fizazi K, Ivanov S, Karlsson CT, Flechon A, Skoneczna I, et al. Aflibercept versus placebo in combination with docetaxel and prednisone for treatment of men with metastatic castration-resistant prostate cancer (VENICE): a phase 3, dou-ble-blind randomised trial. Lancet Oncol. 2013;14:760-8.

395. Taplin ME, Montgomery B, Logothetis CJ, Bubley GJ, Richie JP, Dalkin BL, et al. Intense androgen-deprivation therapy with abiraterone acetate plus leuprolide acetate in patients with localized high-risk prostate cancer: results of a randomized phase II neoad-juvant study. J Clin Oncol. 2014;32:3705-15.

396. Tarbell NJ, Friedman H, Polkinghorn WR, Yock T, Zhou T, Chen Z, et al. High-risk medulloblastoma: a pediatric oncology group randomized trial of chemothera-py before or after radiation therapy (POG 9031). J Clin Oncol. 2013;31:2936-41.

397. Tevaarwerk AJ, Wang M, Zhao F, Fetting JH, Cella D, Wagner LI, et al. Phase III comparison of tamoxifen versus tamoxifen plus ovarian function suppression in premenopausal women with node-negative, hormone receptor-positive breast cancer (E-3193, INT-0142): a trial of the Eastern Cooperative Oncology Group. J Clin Oncol. 2014;32:3948-58.

398. Tewari KS, Sill MW, Long HJ, 3rd, Penson RT, Huang H, Ramondetta LM, et al. Improved survival with bevacizumab in advanced cervical cancer. N Engl J Med. 2014;370:734-43.

399. Thatcher N, Hirsch FR, Luft AV, Szczesna A, Ciuleanu TE, Dediu M, et al. Nec-itumumab plus gemcitabine and cisplatin versus gemcitabine and cisplatin alone as first-line therapy in patients with stage IV squamous non-small-cell lung cancer (SQUIRE): an open-label, randomised, controlled phase 3 trial. Lancet Oncol. 2015;16:763-74.

400. Topal B, Fieuws S, Aerts R, Weerts J, Feryn T, Roeyen G, et al. Pancreaticojeju-nostomy versus pancreaticogastrostomy reconstruction after pancreaticoduodenectomy for pancreatic or periampullary tumours: a multicentre randomised trial. Lancet Oncol. 2013;14:655-62.

401. Tournigand C, Chibaudel B, Samson B, Scheithauer W, Vernerey D, Mesange P, et al. Bevacizumab with or without erlotinib as maintenance therapy in patients with metastatic colorectal cancer (GERCOR DREAM; OPTIMOX3): a randomised, open-label, phase 3 trial. Lancet Oncol. 2015;16:1493-505.

402. Tsuburaya A, Yoshida K, Kobayashi M, Yoshino S, Takahashi M, Takiguchi N, et al. Sequential paclitaxel followed by tegafur and uracil (UFT) or S-1 versus UFT or S-1 monotherapy as adjuvant chemotherapy for T4a/b gastric cancer (SAMIT): a phase 3 fac-torial randomised controlled trial. Lancet Oncol. 2014;15:886-93.

403. Turner NC, Ro J, Andre F, Loi S, Verma S, Iwata H, et al. Palbociclib in Hor-mone-Receptor-Positive Advanced Breast Cancer. N Engl J Med. 2015;373:209-19.

404. Ueno H, Ioka T, Ikeda M, Ohkawa S, Yanagimoto H, Boku N, et al. Randomized phase III study of gemcitabine plus S-1, S-1 alone, or gemcitabine alone in patients with locally advanced and metastatic pancreatic cancer in Japan and Taiwan: GEST study. J Clin Oncol. 2013;31:1640-8.

405. Valle JW, Wasan H, Lopes A, Backen AC, Palmer DH, Morris K, et al. Cediranib or placebo in combination with cisplatin and gemcitabine chemotherapy for patients with advanced biliary tract cancer (ABC-03): a randomised phase 2 trial. Lancet Oncol. 2015;16:967-78.

406. van den Berg SW, Gielissen MF, Custers JA, van der Graaf WT, Ottevanger PB, Prins JB. BREATH: Web-Based Self-Management for Psychological Adjustment After Pri-mary Breast Cancer--Results of a Multicenter Randomized Controlled Trial. J Clin Oncol. 2015;33:2763-71.

407. van Rhee F, Wong RS, Munshi N, Rossi JF, Ke XY, Fossa A, et al. Siltuximab for multicentric Castleman's disease: a randomised, double-blind, placebo-controlled trial. Lancet Oncol. 2014;15:966-74.

408. van Waart H, Stuiver MM, van Harten WH, Geleijn E, Kieffer JM, Buffart LM, et al. Effect of Low-Intensity Physical Activity and Moderate- to High-Intensity Physical Ex-ercise During Adjuvant Chemotherapy on Physical Fitness, Fatigue, and Chemotherapy Completion Rates: Results of the PACES Randomized Clinical Trial. J Clin Oncol. 2015;33:1918-27.

409. Vansteenkiste J, Zielinski M, Linder A, Dahabreh J, Gonzalez EE, Malinowski W, et al. Adjuvant MAGE-A3 immunotherapy in resected non-small-cell lung cancer: phase II randomized study results. J Clin Oncol. 2013;31:2396-403.

410. Vergote IB, Chekerov R, Amant F, Harter P, Casado A, Emerich J, et al. Ran-domized, phase II, placebo-controlled, double-blind study with and without enzastaurin in combination with paclitaxel and carboplatin as first-line treatment followed by maintenance treatment in advanced ovarian cancer. J Clin Oncol. 2013;31:3127-32.

411. Vergote IB, Garcia A, Micha J, Pippitt C, Bendell J, Spitz D, et al. Randomized multicenter phase II trial comparing two schedules of etirinotecan pegol (NKTR-102) in women with recurrent platinum-resistant/refractory epithelial ovarian cancer. J Clin On-col. 2013;31:4060-6.

412. Vergote IB, Jimeno A, Joly F, Katsaros D, Coens C, Despierre E, et al. Random-ized phase III study of erlotinib versus observation in patients with no evidence of disease progression after first-line platin-based chemotherapy for ovarian carcinoma: a European Organisation for Research and Treatment of Cancer-Gynaecological Cancer Group, and Gynecologic Cancer Intergroup study. J Clin Oncol. 2014;32:320-6.

413. Vermorken JB, Stohlmacher-Williams J, Davidenko I, Licitra L, Winquist E, Vil-lanueva C, et al. Cisplatin and fluorouracil with or without panitumumab in patients with recurrent or metastatic squamous-cell carcinoma of the head and neck (SPECTRUM): an open-label phase 3 randomised trial. Lancet Oncol. 2013;14:697-710.

414. Veronesi U, Orecchia R, Maisonneuve P, Viale G, Rotmensz N, Sangalli C, et al. Intraoperative radiotherapy versus external radiotherapy for early breast cancer (ELIOT): a randomised controlled equivalence trial. Lancet Oncol. 2013;14:1269-77.

415. Vitolins MZ, Griffin L, Tomlinson WV, Vuky J, Adams PT, Moose D, et al. Randomized trial to assess the impact of venlafaxine and soy protein on hot flashes and quality of life in men with prostate cancer. J Clin Oncol. 2013;31:4092-8.

416. Vitolo U, Ladetto M, Boccomini C, Baldini L, De Angelis F, Tucci A, et al. Rituximab maintenance compared with observation after brief first-line R-FND chemo-immunotherapy with rituximab consolidation in patients age older than 60 years with advanced follicular lymphoma: a phase III randomized study by the Fondazione Italiana Linfomi. J Clin Oncol. 2013;31:3351-9.

417. Von Hoff DD, Ervin T, Arena FP, Chiorean EG, Infante J, Moore M, et al. In-creased survival in pancreatic cancer with nab-paclitaxel plus gemcitabine. N Engl J Med. 2013;369:1691-703.

418. von Minckwitz G, Mobus V, Schneeweiss A, Huober J, Thomssen C, Untch M, et al. German adjuvant intergroup node-positive study: a phase III trial to compare oral ibandronate versus observation in patients with high-risk early breast cancer. J Clin Oncol. 2013;31:3531-9.

419. von Minckwitz G, Puglisi F, Cortes J, Vrdoljak E, Marschner N, Zielinski C, et al. Bevacizumab plus chemotherapy versus chemotherapy alone as second-line treatment for patients with HER2-negative locally recurrent or metastatic breast cancer after first-line treatment with bevacizumab plus chemotherapy (TANIA): an open-label, randomised phase 3 trial. Lancet Oncol. 2014;15:1269-78.

420. von Minckwitz G, Schneeweiss A, Loibl S, Salat C, Denkert C, Rezai M, et al. Neoadjuvant carboplatin in patients with triple-negative and HER2-positive early breast cancer (GeparSixto; GBG 66): a randomised phase 2 trial. Lancet Oncol. 2014;15:747-56.

421. von Pawel J, Jotte R, Spigel DR, O'Brien ME, Socinski MA, Mezger J, et al. Ran-domized phase III trial of amrubicin versus topotecan as second-line treatment for pa-tients with small-cell lung cancer. J Clin Oncol. 2014;32:4012-9.

422. Vora A, Goulden N, Mitchell C, Hancock J, Hough R, Rowntree C, et al. Aug-mented post-remission therapy for a minimal residual disease-defined high-risk sub-group of children and young people with clinical standard-risk and intermediate-risk acute lymphoblastic leukaemia (UKALL 2003): a randomised controlled trial. Lancet On-col. 2014;15:809-18.

423. Vora A, Goulden N, Wade R, Mitchell C, Hancock J, Hough R, et al. Treatment reduction for children and young adults with low-risk acute lymphoblastic leukaemia de-fined by minimal residual disease (UKALL 2003): a randomised controlled trial. Lancet Oncol. 2013;14:199-209.

424. Vose JM, Carter S, Burns LJ, Ayala E, Press OW, Moskowitz CH, et al. Phase III randomized study of rituximab/carmustine, etoposide, cytarabine, and melphalan (BEAM) compared with iodine-131 tositumomab/BEAM with autologous hematopoietic cell transplantation for relapsed diffuse large B-cell lymphoma: results from the BMT CTN 0401 trial. J Clin Oncol. 2013;31:1662-8.

425. Vrooman LM, Stevenson KE, Supko JG, O'Brien J, Dahlberg SE, Asselin BL, et al. Postinduction dexamethasone and individualized dosing of Escherichia Coli L-asparaginase each improve outcome of children and adolescents with newly diagnosed acute lymphoblastic leukemia: results from a randomized study--Dana-Farber Cancer In-stitute ALL Consortium Protocol 00-01. J Clin Oncol. 2013;31:1202-10.

426. Waddell T, Chau I, Cunningham D, Gonzalez D, Okines AF, Okines C, et al. Epirubicin, oxaliplatin, and capecitabine with or without panitumumab for patients with previously untreated advanced oesophagogastric cancer (REAL3): a randomised, open-label phase 3 trial. Lancet Oncol. 2013;14:481-9.

427. Walker I, Panzarella T, Couban S, Couture F, Devins G, Elemary M, et al. Pre-treatment with anti-thymocyte globulin versus no anti-thymocyte globulin in patients with haematological malignancies undergoing haemopoietic cell transplantation from unrelated donors: a randomised, controlled, open-label, phase 3, multicentre trial. Lancet Oncol. 2016;17:164-73.

428. Walker J, Hansen CH, Martin P, Symeonides S, Gourley C, Wall L, et al. Inte-grated collaborative care for major depression comorbid with a poor prognosis cancer (SMaRT Oncology-3): a multicentre randomised controlled trial in patients with lung cancer. Lancet Oncol. 2014;15:1168-76.

429. Wasan H, Meade AM, Adams R, Wilson R, Pugh C, Fisher D, et al. Intermittent chemotherapy plus either intermittent or continuous cetuximab for first-line treatment of patients with KRAS wild-type advanced colorectal cancer (COIN-B): a randomised phase 2 trial. Lancet Oncol. 2014;15:631-9.

430. Weber JS, D'Angelo SP, Minor D, Hodi FS, Gutzmer R, Neyns B, et al. Nivolumab versus chemotherapy in patients with advanced melanoma who progressed after anti-CTLA-4 treatment (CheckMate 037): a randomised, controlled, open-label, phase 3 trial. Lancet Oncol. 2015;16:375-84.

431. Wenzel L, Osann K, Hsieh S, Tucker JA, Monk BJ, Nelson EL. Psychosocial tel-ephone counseling for survivors of cervical cancer: results of a randomized biobehavioral trial. J Clin Oncol. 2015;33:1171-9.

432. Westphal M, Yla-Herttuala S, Martin J, Warnke P, Menei P, Eckland D, et al. Adenovirus-mediated gene therapy with sitimagene ceradenovec followed by intravenous ganciclovir for patients with operable high-grade glioma (ASPECT): a randomised, open-label, phase 3 trial. Lancet Oncol. 2013;14:823-33.

433. Whelan TJ, Olivotto IA, Parulekar WR, Ackerman I, Chua BH, Nabid A, et al. Regional Nodal Irradiation in Early-Stage Breast Cancer. N Engl J Med. 2015;373:307-16.

434. White VM, Young MA, Farrelly A, Meiser B, Jefford M, Williamson E, et al. Randomized controlled trial of a telephone-based peer-support program for women car-rying a BRCA1 or BRCA2 mutation: impact on psychological distress. J Clin Oncol. 2014;32:4073-80.

435. Wilke H, Muro K, Van Cutsem E, Oh SC, Bodoky G, Shimada Y, et al. Ramu-cirumab plus paclitaxel versus placebo plus paclitaxel in patients with previously treated advanced gastric or gastro-oesophageal junction adenocarcinoma (RAINBOW): a dou-ble-blind, randomised phase 3 trial. Lancet Oncol. 2014;15:1224-35.

436. Willemze R, Suciu S, Meloni G, Labar B, Marie JP, Halkes CJ, et al. High-dose cytarabine in induction treatment improves the outcome of adult patients younger than age 46 years with acute myeloid leukemia: results of the EORTC-GIMEMA AML-12 trial. J Clin Oncol. 2014;32:219-28.

437. Wislez M, Barlesi F, Besse B, Mazieres J, Merle P, Cadranel J, et al. Customized adjuvant phase II trial in patients with non-small-cell lung cancer: IFCT-0801 TASTE. J Clin Oncol. 2014;32:1256-61.

438. Wolfe J, Orellana L, Cook EF, Ullrich C, Kang T, Geyer JR, et al. Improving the care of children with advanced cancer by using an electronic patient-reported feedback intervention: results from the PediQUEST randomized controlled trial. J Clin Oncol. 2014;32:1119-26.

439. Wolff AC, Lazar AA, Bondarenko I, Garin AM, Brincat S, Chow L, et al. Ran-domized phase III placebo-controlled trial of letrozole plus oral temsirolimus as first-line endocrine therapy in postmenopausal women with locally advanced or metastatic breast cancer. J Clin Oncol. 2013;31:195-202.

440. Wu YL, Lee JS, Thongprasert S, Yu CJ, Zhang L, Ladrera G, et al. Intercalated combination of chemotherapy and erlotinib for patients with advanced stage non-small-cell lung cancer (FASTACT-2): a randomised, double-blind trial. Lancet Oncol. 2013;14:777-86.

441. Wu YL, Zhou C, Hu CP, Feng J, Lu S, Huang Y, et al. Afatinib versus cisplatin plus gemcitabine for first-line treatment of Asian patients with advanced non-small-cell lung cancer harbouring EGFR mutations (LUX-Lung 6): an open-label, randomised phase 3 trial. Lancet Oncol. 2014;15:213-22.

442. Yamada Y, Takahari D, Matsumoto H, Baba H, Nakamura M, Yoshida K, et al. Leucovorin, fluorouracil, and oxaliplatin plus bevacizumab versus S-1 and oxaliplatin plus bevacizumab in patients with metastatic colorectal cancer (SOFT): an open-label, non-inferiority, randomised phase 3 trial. Lancet Oncol. 2013;14:1278-86.

443. Yardley DA, Ismail-Khan RR, Melichar B, Lichinitser M, Munster PN, Klein PM, et al. Randomized phase II, double-blind, placebo-controlled study of exemestane with or without entinostat in postmenopausal women with locally recurrent or metastatic estrogen receptor-positive breast cancer progressing on treatment with a nonsteroidal aromatase inhibitor. J Clin Oncol. 2013;31:2128-35.

444. Yardley DA, Weaver R, Melisko ME, Saleh MN, Arena FP, Forero A, et al. EMERGE: A Randomized Phase II Study of the Antibody-Drug Conjugate Glembatu-mumab Vedotin in Advanced Glycoprotein NMB-Expressing Breast Cancer. J Clin Oncol. 2015;33:1609-19.

445. Ye LC, Liu TS, Ren L, Wei Y, Zhu DX, Zai SY, et al. Randomized controlled trial of cetuximab plus chemotherapy for patients with KRAS wild-type unresectable colorectal liver-limited metastases. J Clin Oncol. 2013;31:1931-8.

446. Yennurajalingam S, Frisbee-Hume S, Palmer JL, Delgado-Guay MO, Bull J, Phan AT, et al. Reduction of cancer-related fatigue with dexamethasone: a double-blind, randomized, placebo-controlled trial in patients with advanced cancer. J Clin Oncol. 2013;31:3076-82.

447. Yu EY, Li H, Higano CS, Agarwal N, Pal SK, Alva A, et al. SWOG S0925: A Randomized Phase II Study of Androgen Deprivation Combined With Cixutumumab Versus Androgen Deprivation Alone in Patients With New Metastatic Hormone-Sensitive Prostate Cancer. J Clin Oncol. 2015;33:1601-8.

448. Zapatero A, Guerrero A, Maldonado X, Alvarez A, Gonzalez San Segundo C, Cabeza Rodriguez MA, et al. High-dose radiotherapy with short-term or long-term an-drogen deprivation in localised prostate cancer (DART01/05 GICOR): a randomised, con-trolled, phase 3 trial. Lancet Oncol. 2015;16:320-7.

449. Zhong LP, Zhang CP, Ren GX, Guo W, William WN, Jr., Sun J, et al. Random-ized phase III trial of induction chemotherapy with docetaxel, cisplatin, and fluorouracil followed by surgery versus up-front surgery in locally advanced resectable oral squamous cell carcinoma. J Clin Oncol. 2013;31:744-51.

450. Zhou C, Wu YL, Chen G, Liu X, Zhu Y, Lu S, et al. BEYOND: A Randomized, Double-Blind, Placebo-Controlled, Multicenter, Phase III Study of First-Line Car-boplatin/Paclitaxel Plus Bevacizumab or Placebo in Chinese Patients With Advanced or Recurrent Nonsquamous Non-Small-Cell Lung Cancer. J Clin Oncol. 2015;33:2197-204.

451. Zhu AX, Park JO, Ryoo BY, Yen CJ, Poon R, Pastorelli D, et al. Ramucirumab versus placebo as second-line treatment in patients with advanced hepatocellular carci-noma following first-line therapy with sorafenib (REACH): a randomised, double-blind, multicentre, phase 3 trial. Lancet Oncol. 2015;16:859-70.

452. Zhu AX, Rosmorduc O, Evans TR, Ross PJ, Santoro A, Carrilho FJ, et al. SEARCH: a phase III, randomized, double-blind, placebo-controlled trial of sorafenib plus erlotinib in patients with advanced hepatocellular carcinoma. J Clin Oncol. 2015;33:559-66.

453. Zhu HD, Guo JH, Mao AW, Lv WF, Ji JS, Wang WH, et al. Conventional stents versus stents loaded with (125)iodine seeds for the treatment of unresectable oesophageal cancer: a multicentre, randomised phase 3 trial. Lancet Oncol. 2014;15:612-9.

454. Zhu HH, Wu DP, Jin J, Li JY, Ma J, Wang JX, et al. Oral tetra-arsenic tetra-sulfide formula versus intravenous arsenic trioxide as first-line treatment of acute promyelocytic leukemia: a multicenter randomized controlled trial. J Clin Oncol. 2013;31:4215-21.

455. Zukin M, Barrios CH, Pereira JR, Ribeiro Rde A, Beato CA, do Nascimento YN, et al. Randomized phase III trial of single-agent pemetrexed versus carboplatin and pemetrexed in patients with advanced non-small-cell lung cancer and Eastern Coopera-tive Oncology Group performance status of 2. J Clin Oncol. 2013;31:2849-53.

456. Zuliani AC, Esteves SC, Teixeira LC, Teixeira JC, de Souza GA, Sarian LO. Concomitant cisplatin plus radiotherapy and high-dose-rate brachytherapy versus radio-therapy alone for stage IIIB epidermoid cervical cancer: a randomized controlled trial. J Clin Oncol. 2014;32:542-7.
